# Supplementary figures and images for: The peroxisomal exportomer directly inhibits phosphoactivation of the pexophagy receptor Atg36 to suppress pexophagy in yeast
Source: eLife. 2022 Apr 11;11:e74531. doi: 10.7554/eLife.74531 (PMC9000956; doi:10.7554/eLife.74531)

Figure 1A

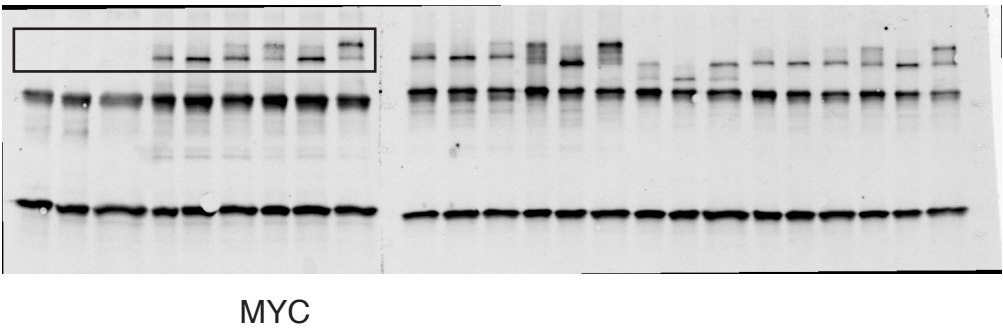

Figure 1B

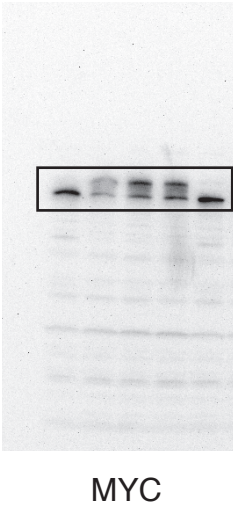

Figure 1C

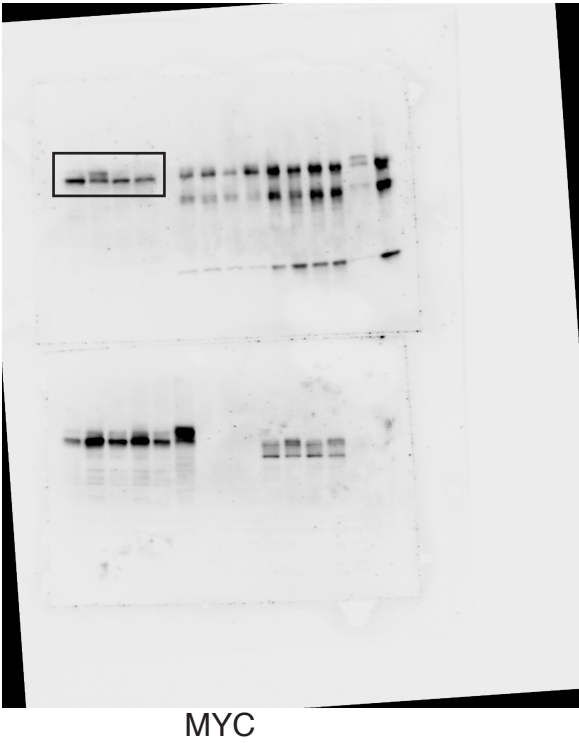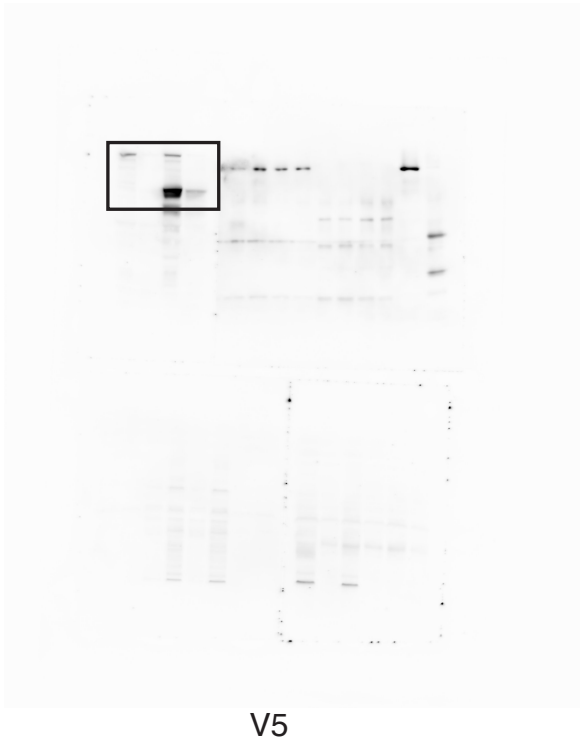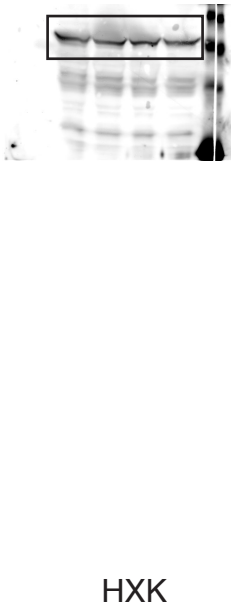

Figure 1D

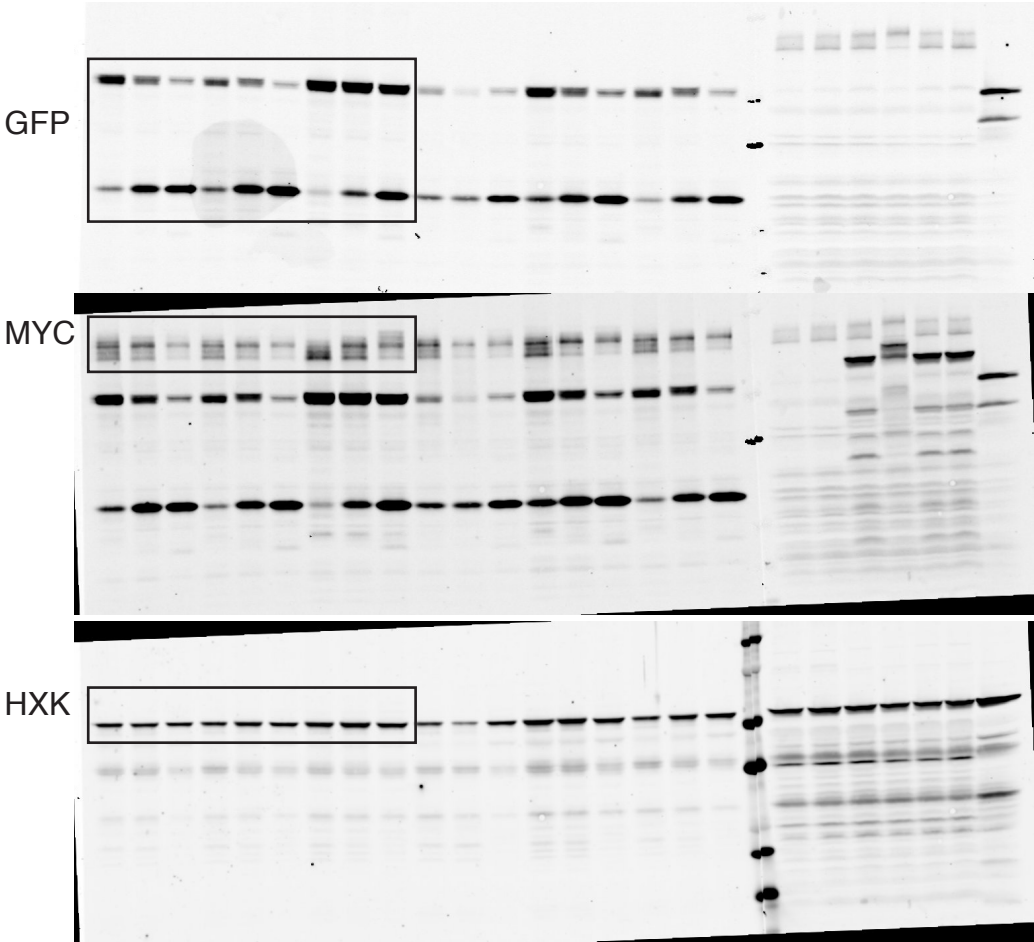

Supplement: Figure 1—source data 1. [file elife-74531-fig1-data1.pdf]

Figure 2A

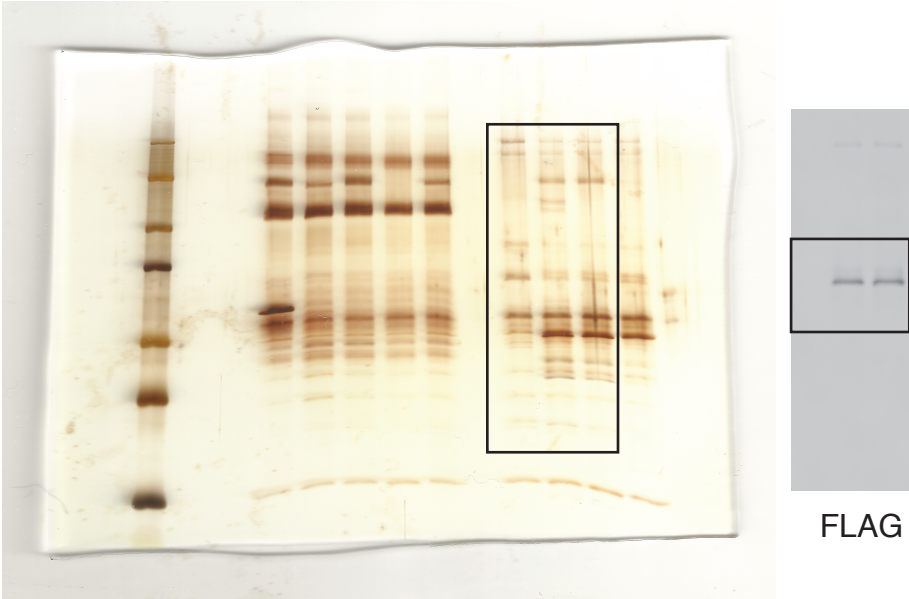

Figure 2B

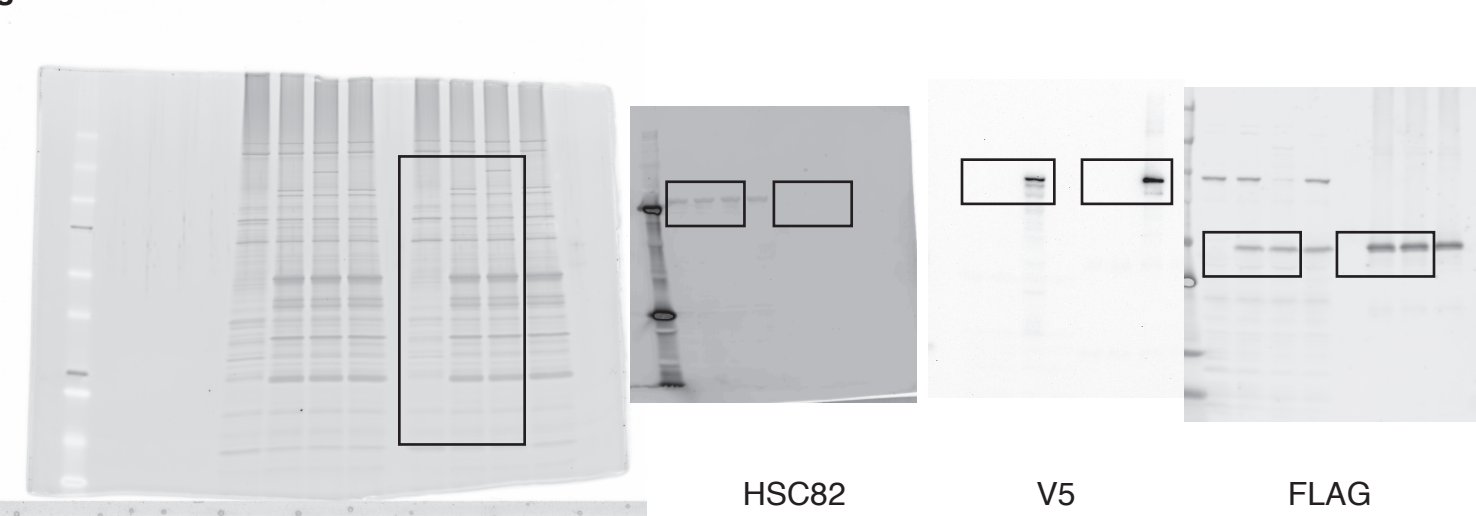

Figure 2C

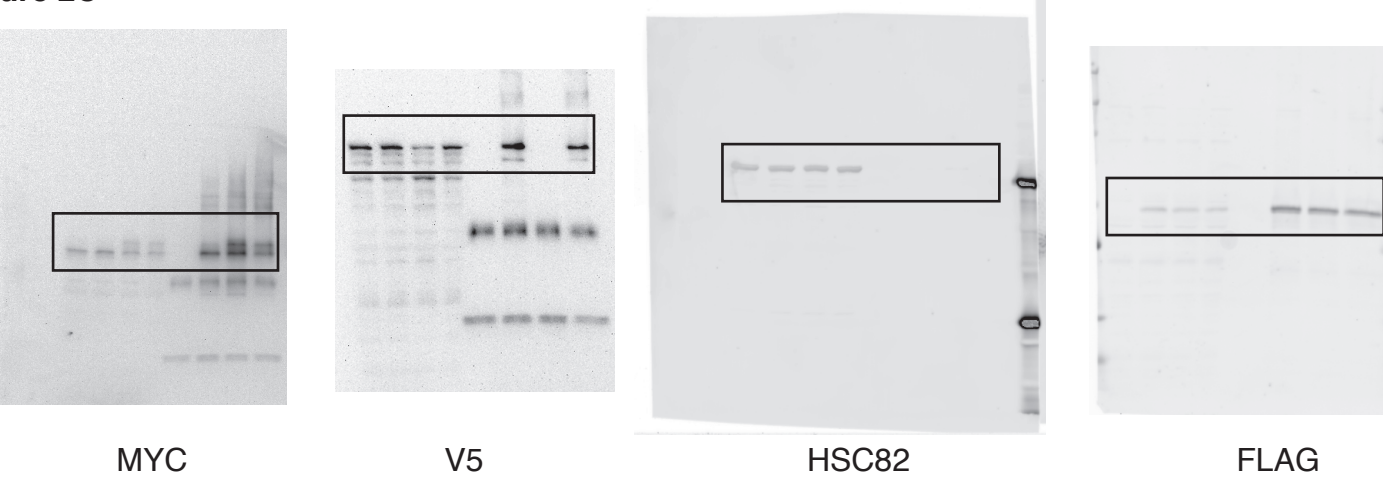

Supplement: Figure 2—source data 1. [file elife-74531-fig2-data1.pdf]

Figure 3A

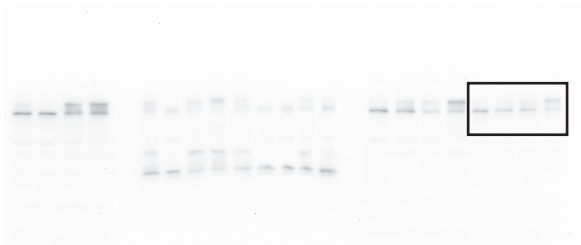

MYC

Figure 3B

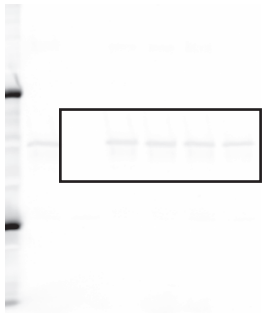

FLAG

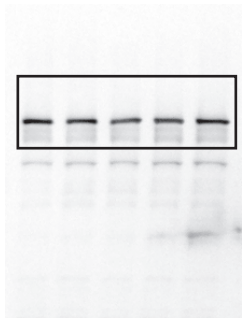

V5 (input)

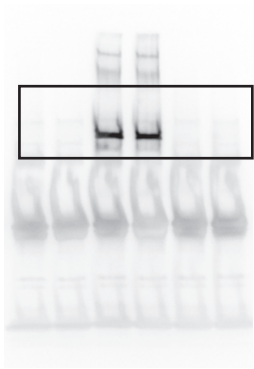

V5 (IP)

Figure 3C

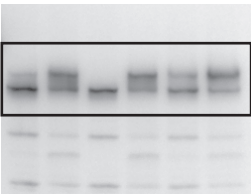

MYC

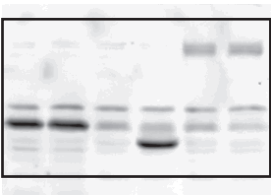

FLAG

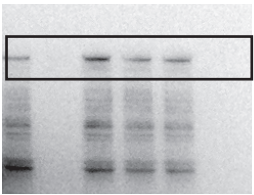

V5

Figure 3E

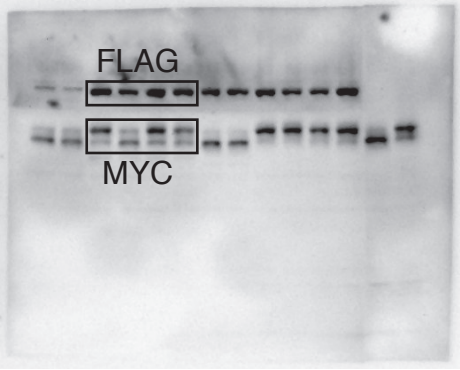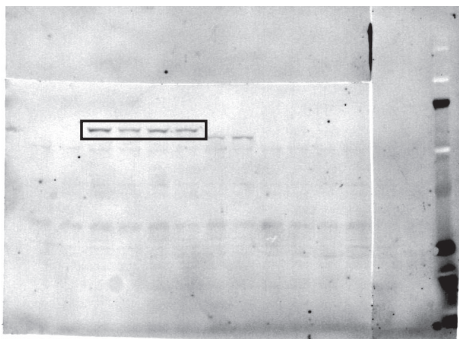

HA

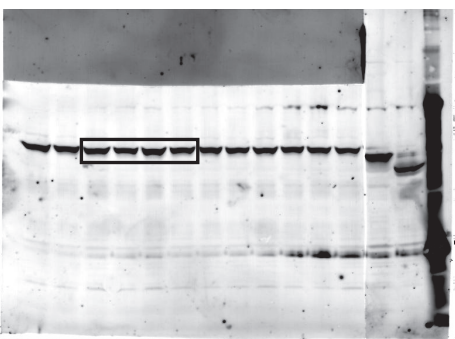

HXK

Figure 3F

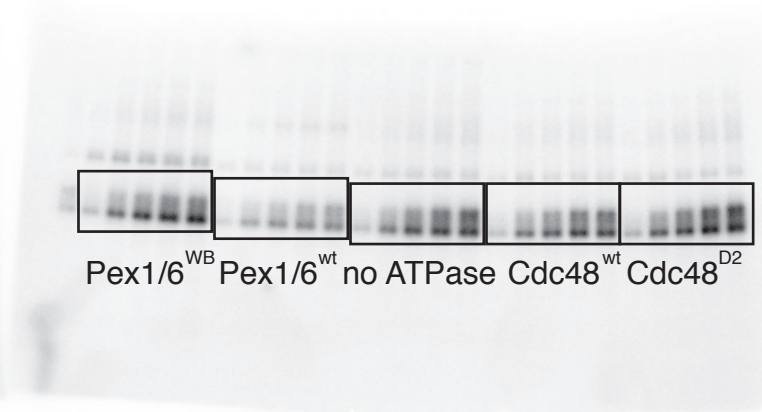

Figure 3G

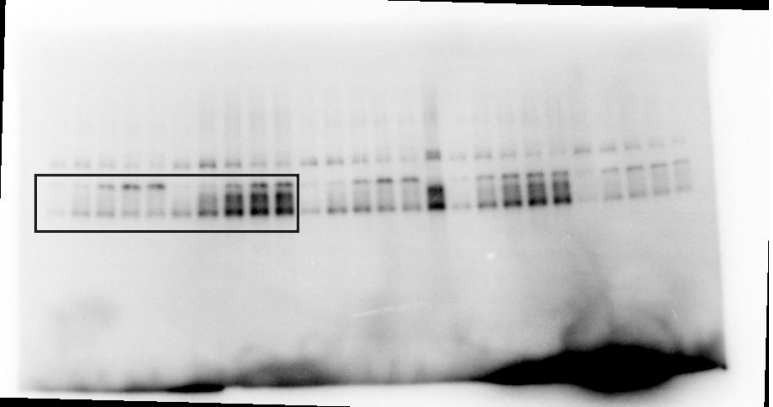

Supplement: Figure 3—source data 1. [file elife-74531-fig3-data1.pdf]

Figure 4C

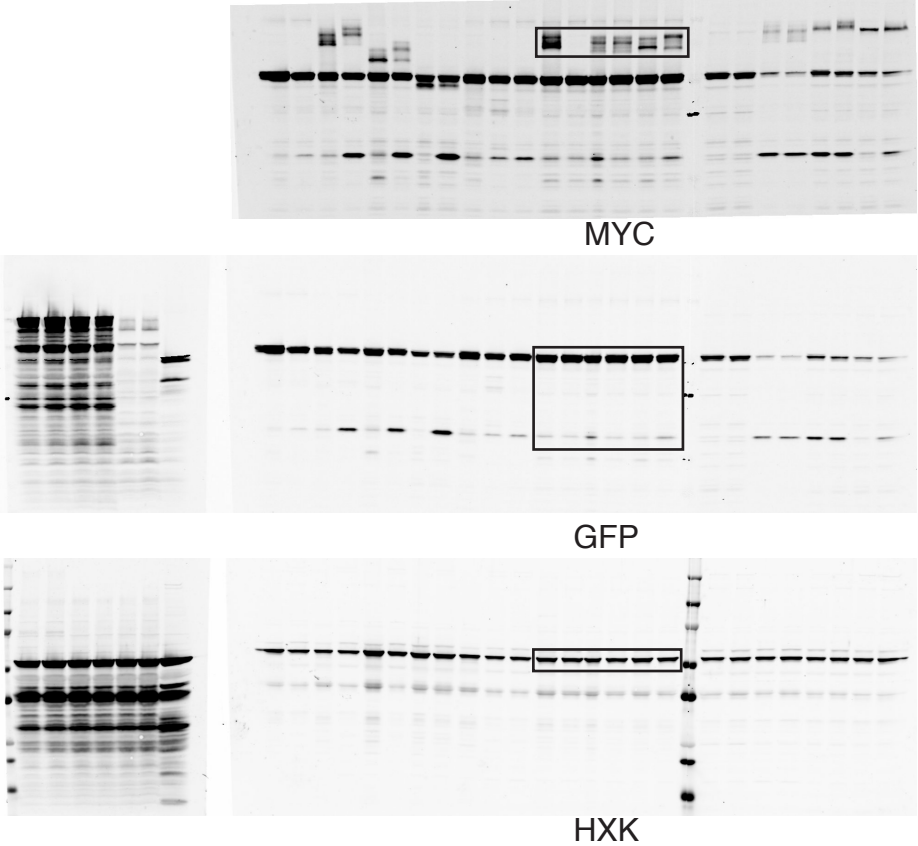

Supplement: Figure 4—source data 1. [file elife-74531-fig4-data1.pdf]

Figure 3-FS2A

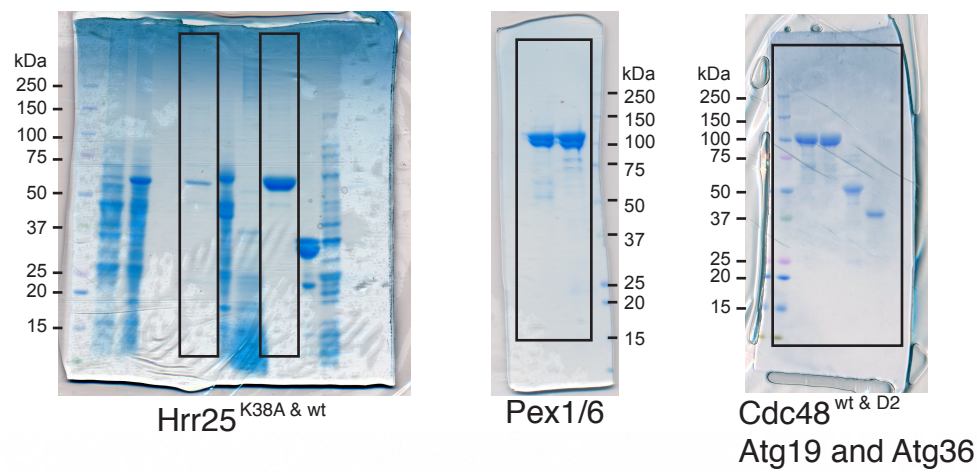

Figure 3-FS2B

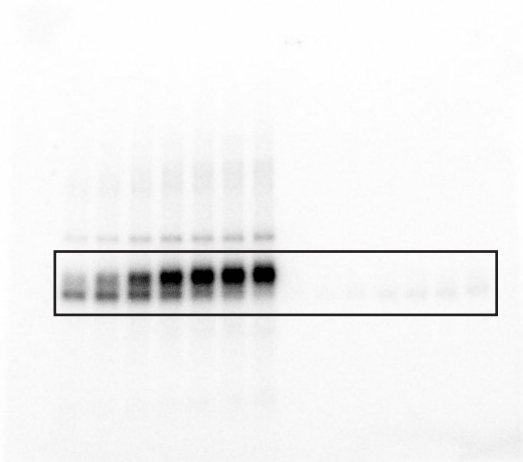

Figure 3-FS2C

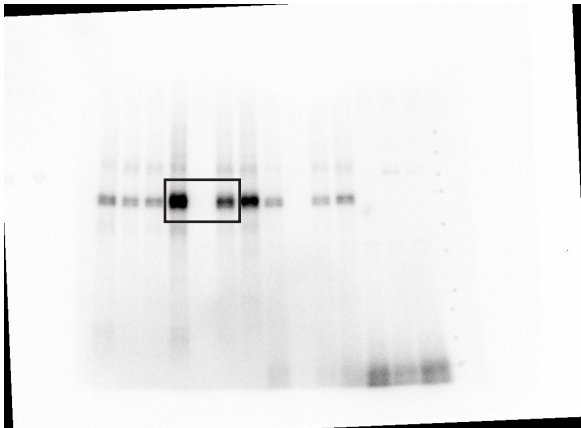

Figure 3-FS2E

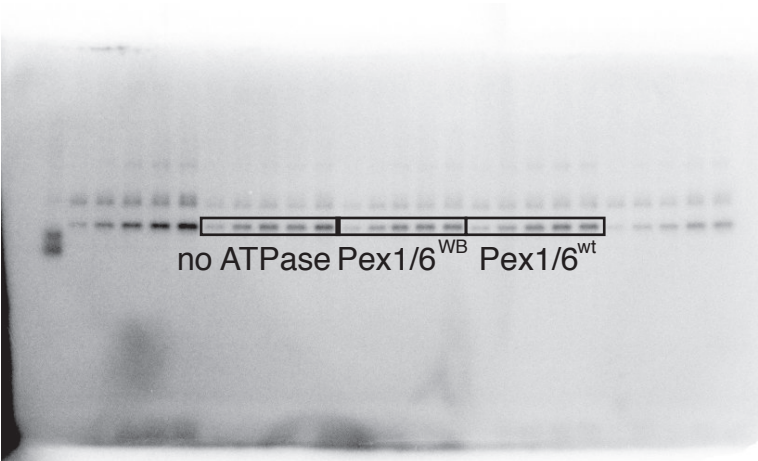

Supplement: Source data 1. [file elife-74531-data1.zip › source data/source data Figure 3-figure supplement 2.pdf]

Figure 4-FS1D

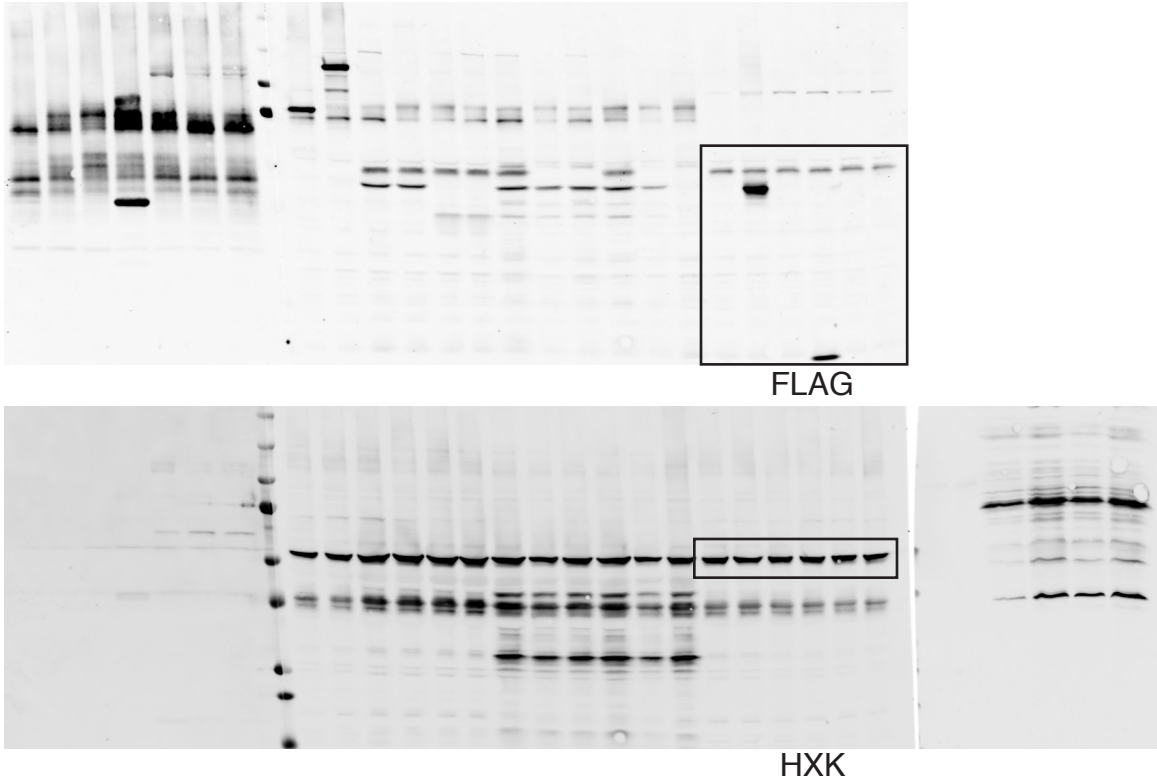

Figure 4-FS1E

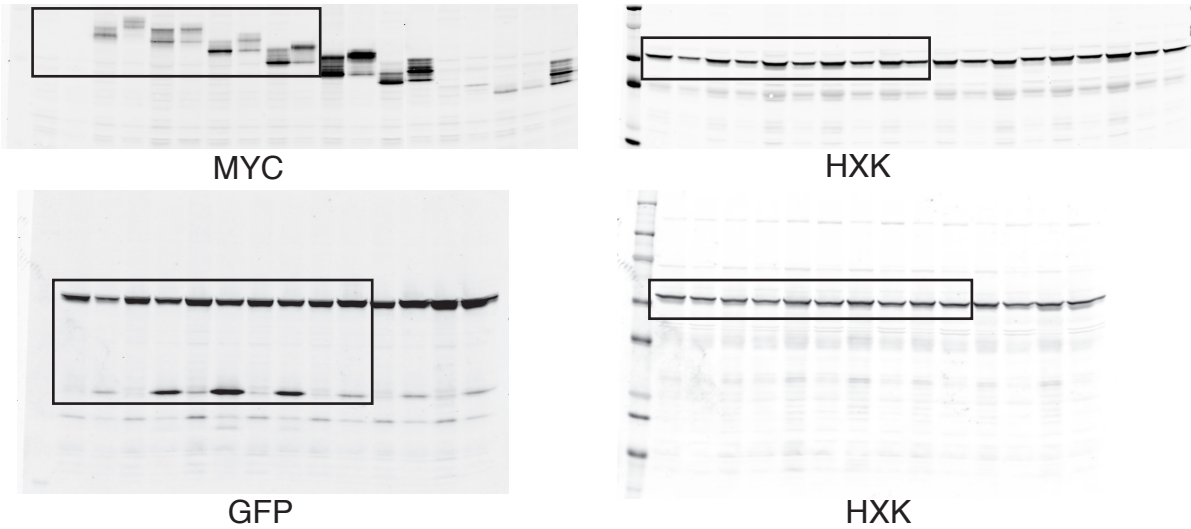

Figure 4-FS1F

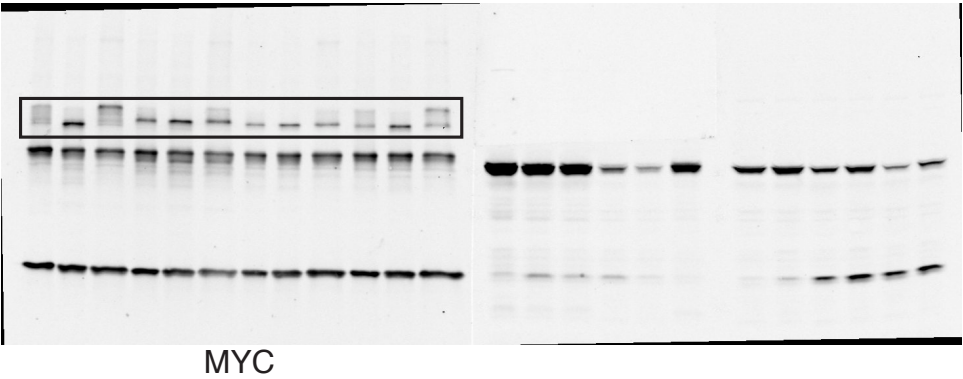

Supplement: Source data 1. [file elife-74531-data1.zip › source data/source data Figure 4-figure supplement 1.pdf]

Figure 1-FS1A

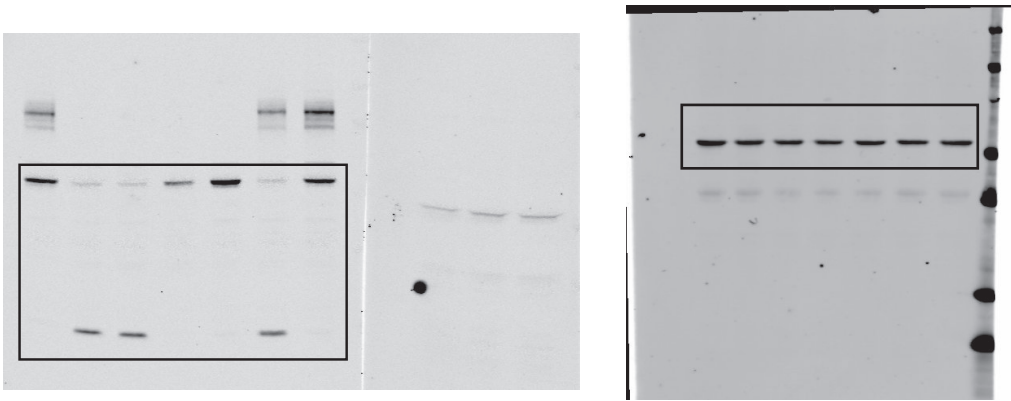

GFP

HXK

Figure 1-FS1B

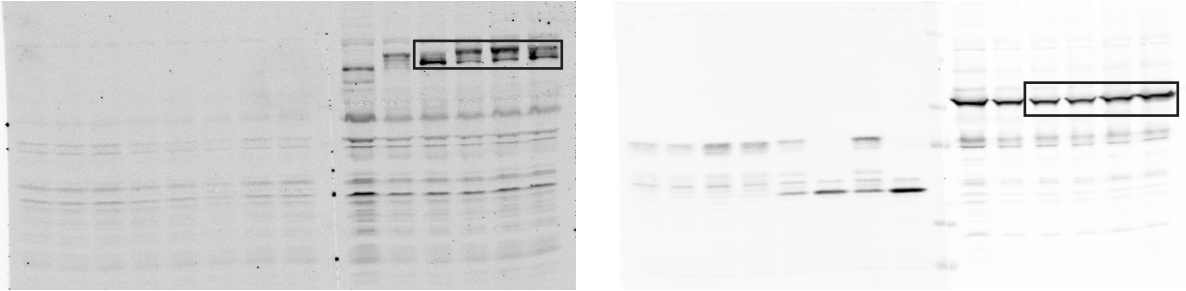

MYC

HXK

Supplement: Source data 1. [file elife-74531-data1.zip › source data/source data Figure 1-figure supplement 1.pdf]

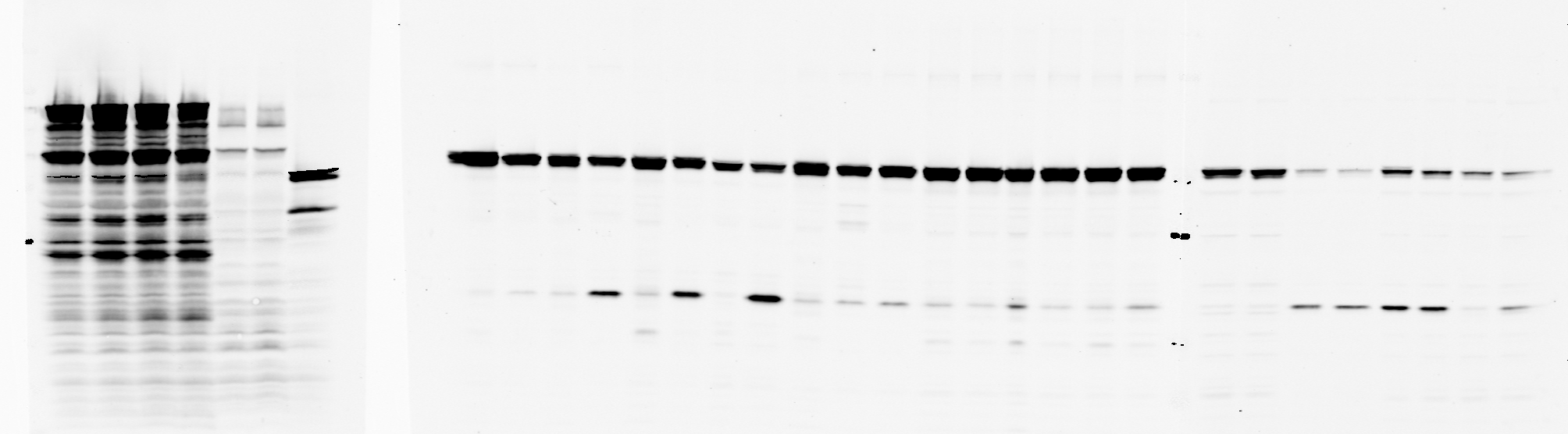

Supplement: Source data 2. [file elife-74531-data2.zip › Source Data Figures/Source Data Figure 4C/Figure 4C GFP.TIF]

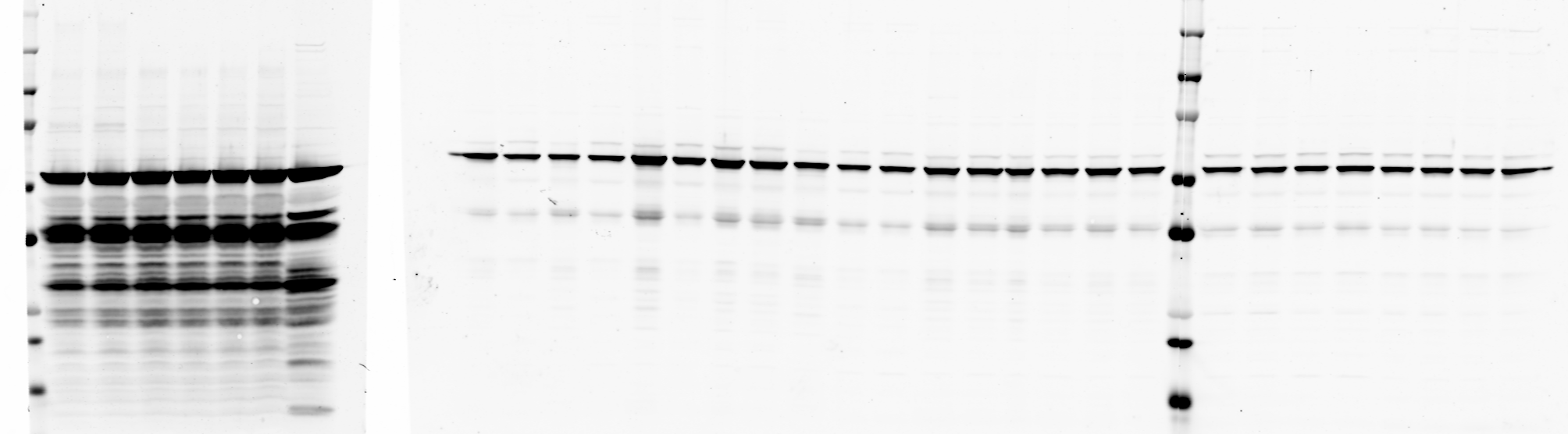

Supplement: Source data 2. [file elife-74531-data2.zip › Source Data Figures/Source Data Figure 4C/Figure 4C HXK.TIF]

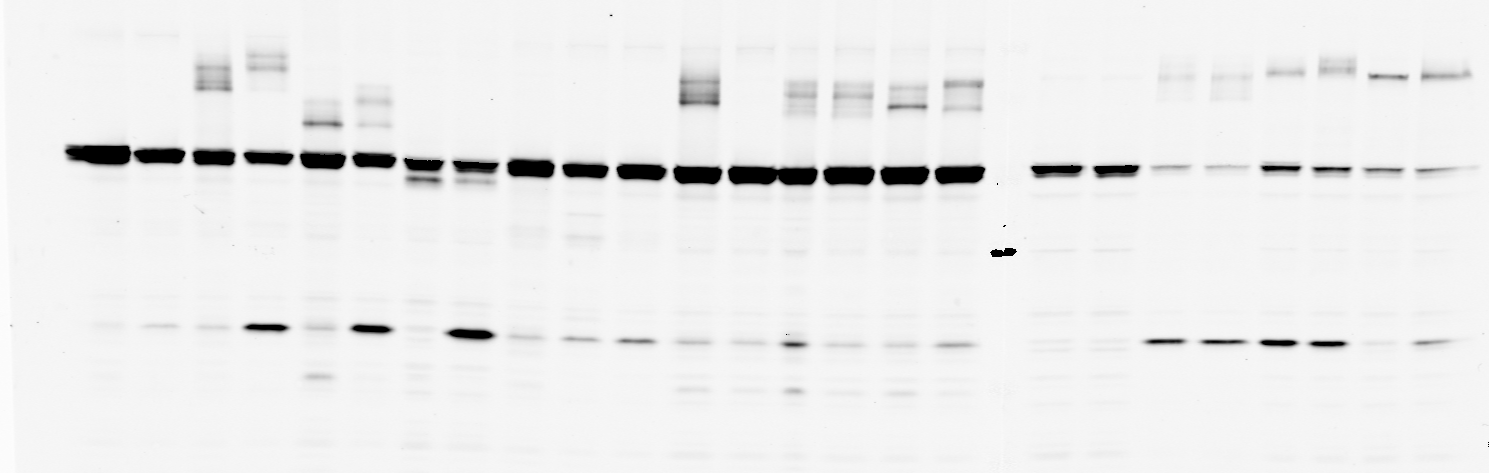

Supplement: Source data 2. [file elife-74531-data2.zip › Source Data Figures/Source Data Figure 4C/Figure 4C MYC.TIF]

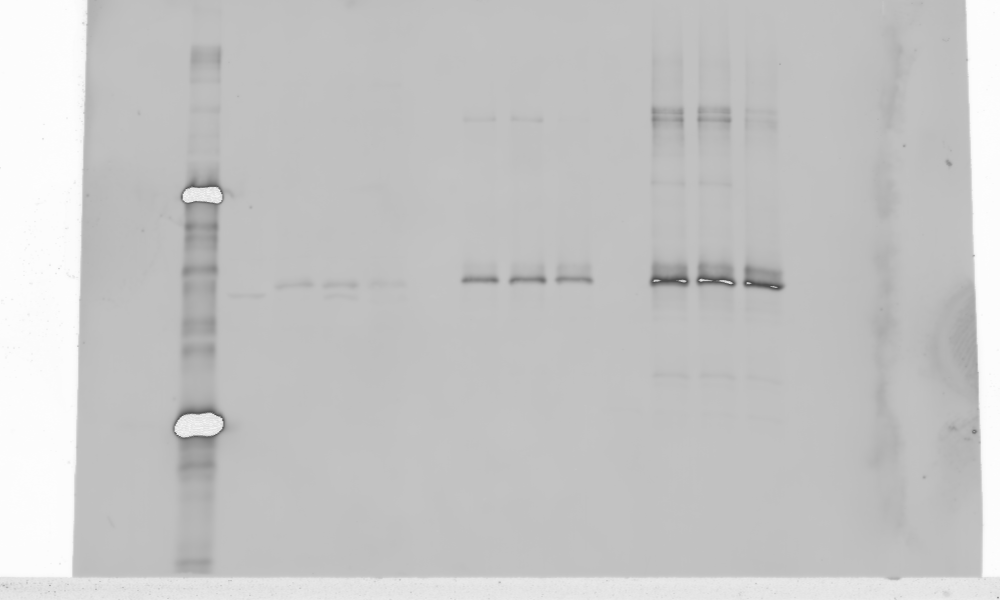

Supplement: Source data 2. [file elife-74531-data2.zip › Source Data Figures/Source Data Figure 2A/Fig2A FLAG.tif]

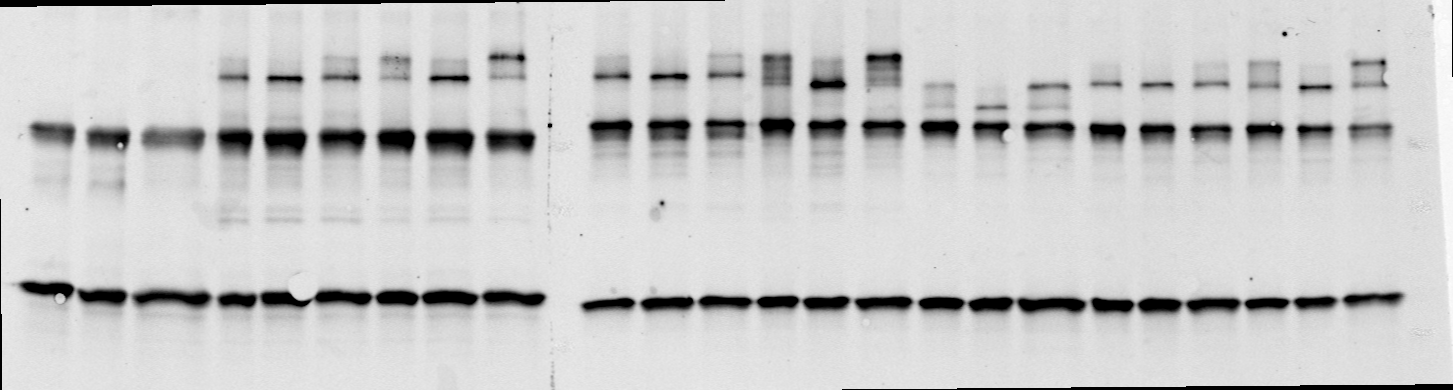

Supplement: Source data 2. [file elife-74531-data2.zip › Source Data Figures/Source Data Figure 1A/Fig 1A MYC.TIF]

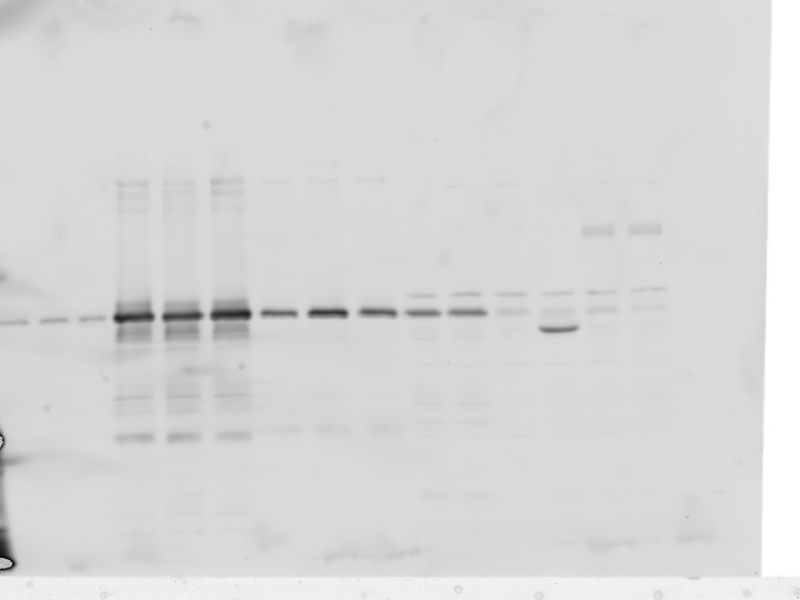

Supplement: Source data 2. [file elife-74531-data2.zip › Source Data Figures/Source Data Figure 3C/Fig3C flag .tiff]

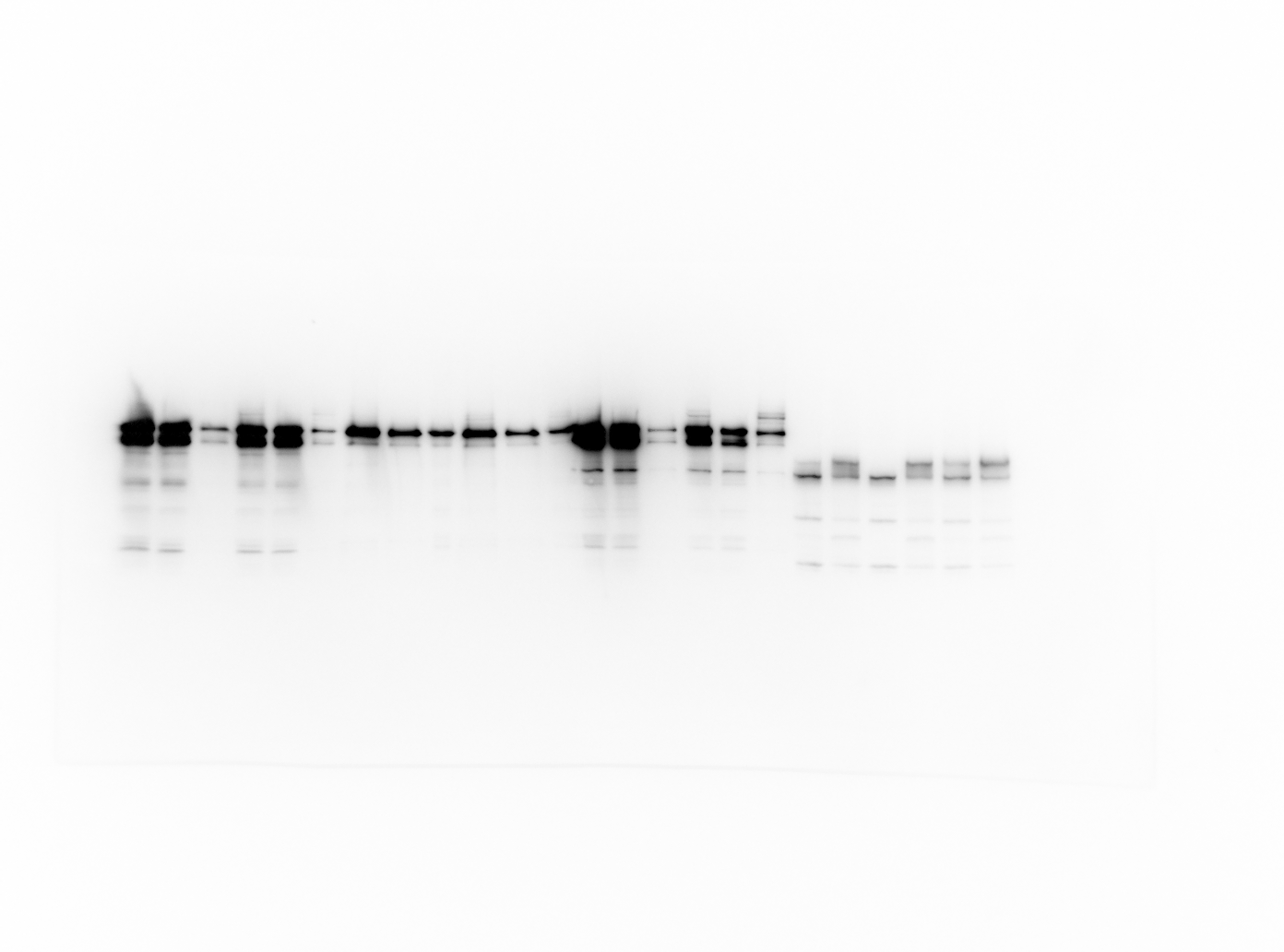

Supplement: Source data 2. [file elife-74531-data2.zip › Source Data Figures/Source Data Figure 3C/Fig3C myc.tif]

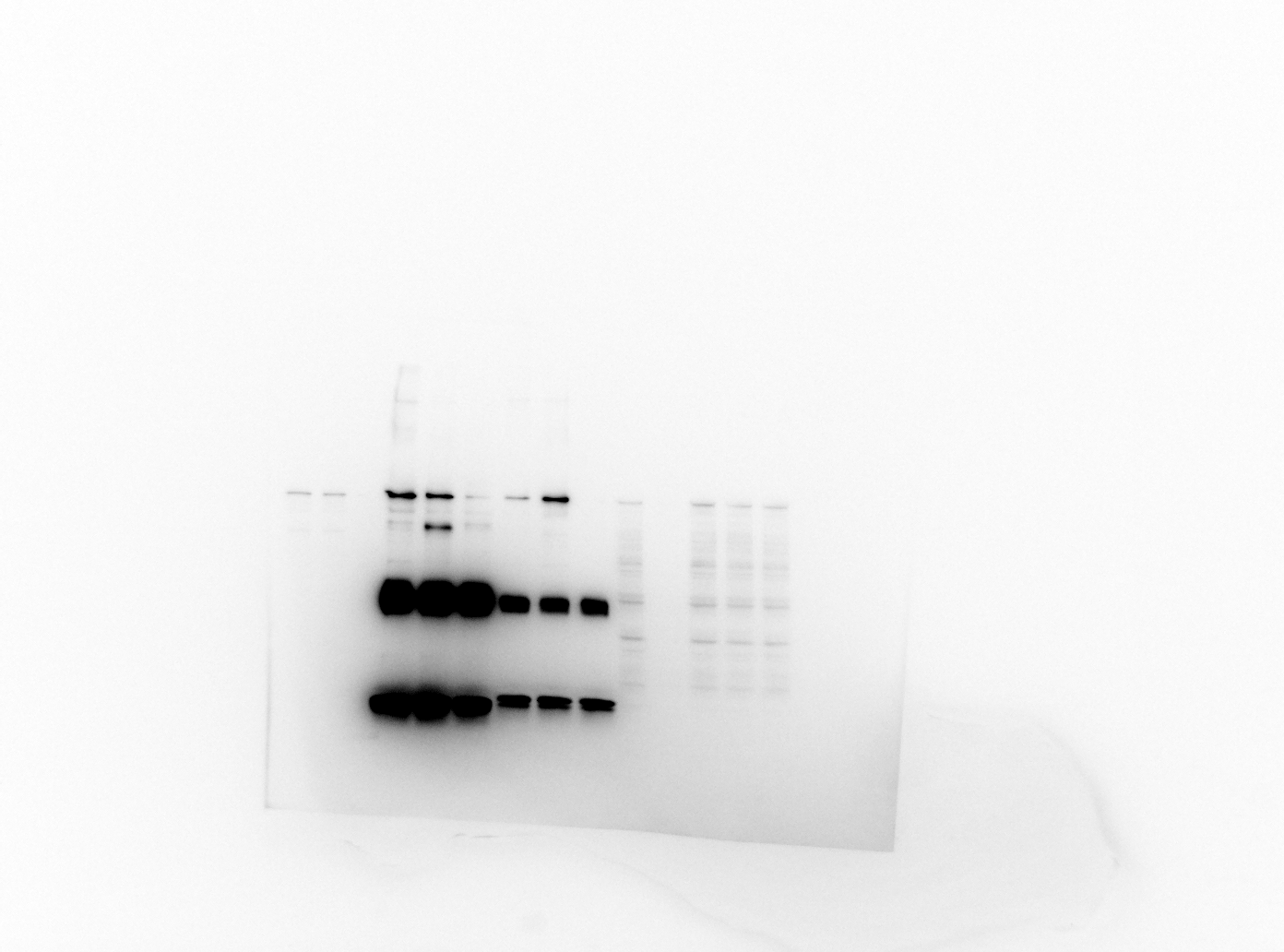

Supplement: Source data 2. [file elife-74531-data2.zip › Source Data Figures/Source Data Figure 3C/Fig3C v5.tif]

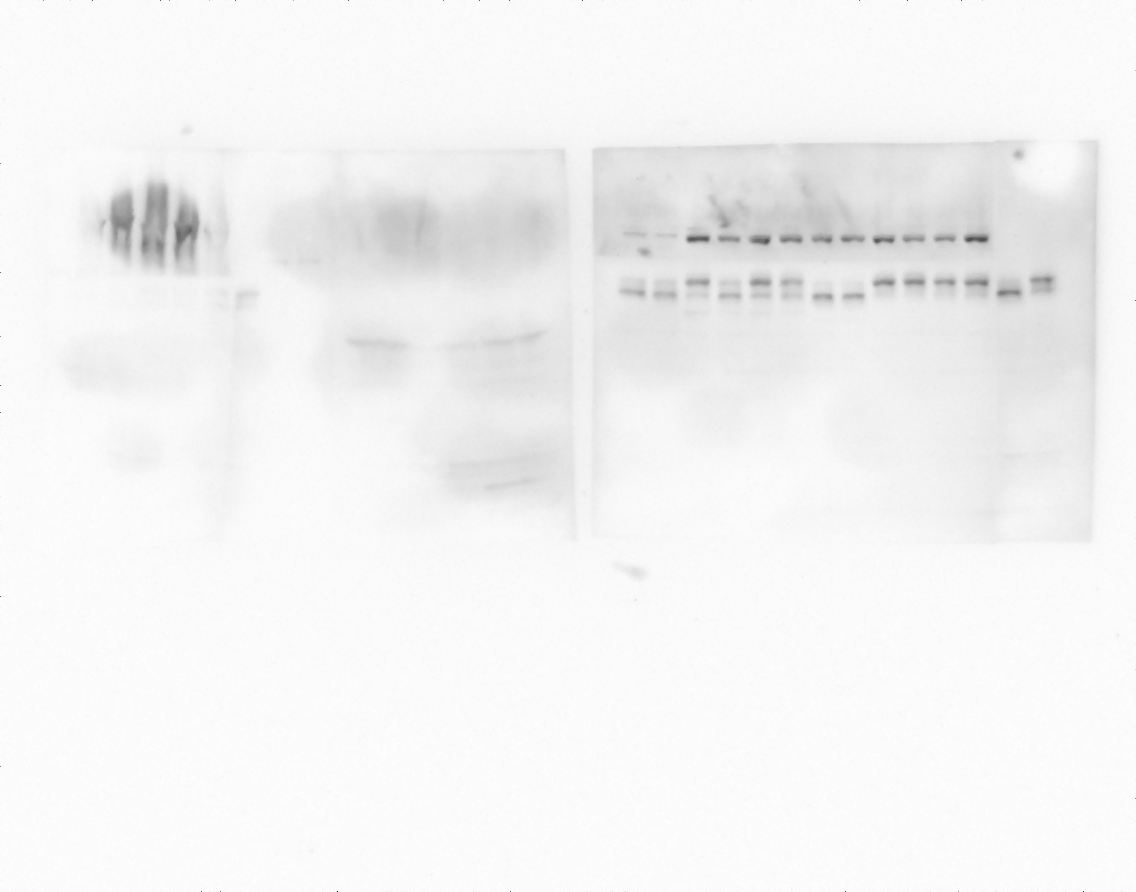

Supplement: Source data 2. [file elife-74531-data2.zip › Source Data Figures/Source Data Figure 3E/Figure 3E MYC & FLAG.tif]

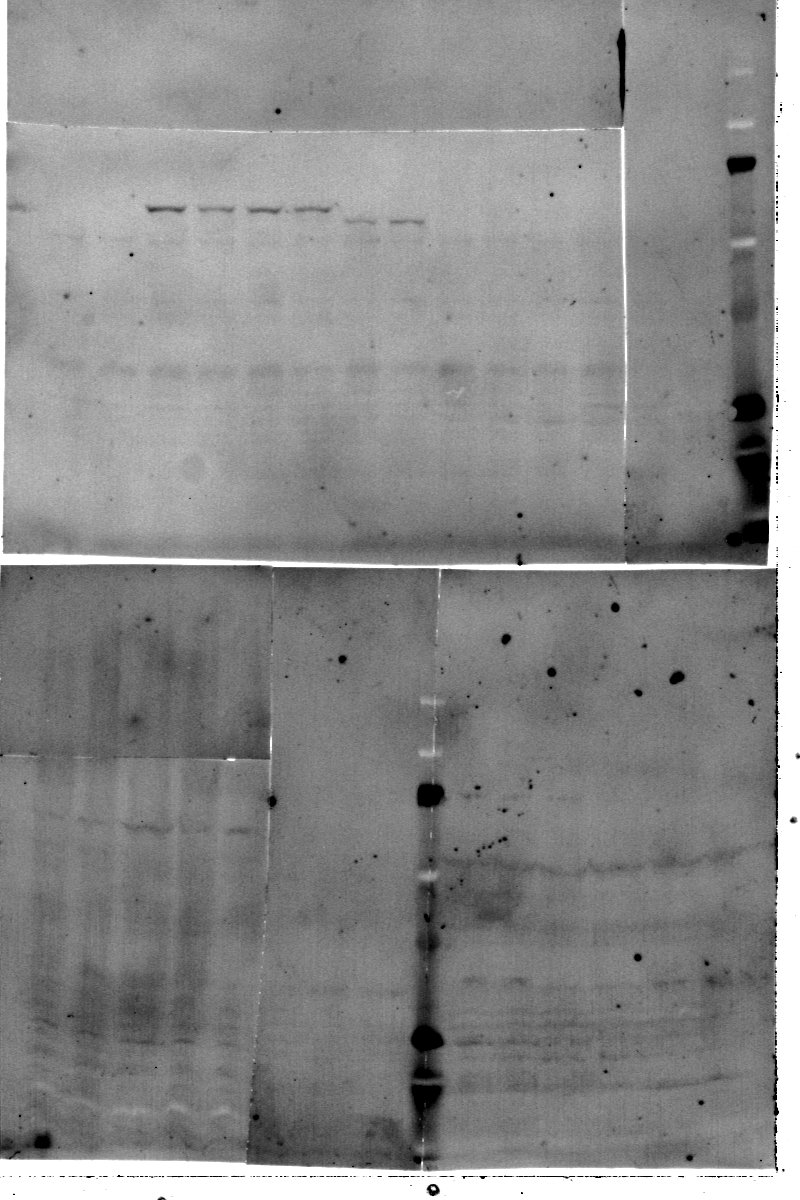

Supplement: Source data 2. [file elife-74531-data2.zip › Source Data Figures/Source Data Figure 3E/Figure 3E HA.tif]

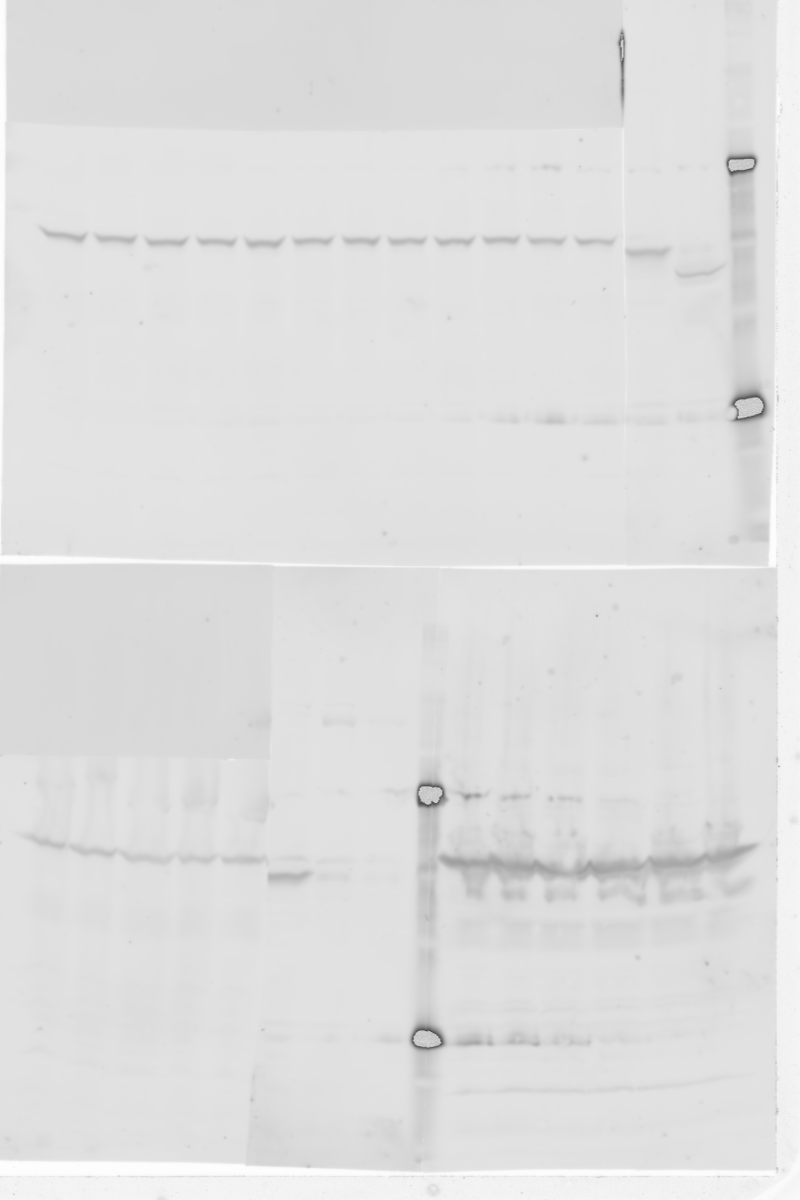

Supplement: Source data 2. [file elife-74531-data2.zip › Source Data Figures/Source Data Figure 3E/Figure 3E HXK.tif]

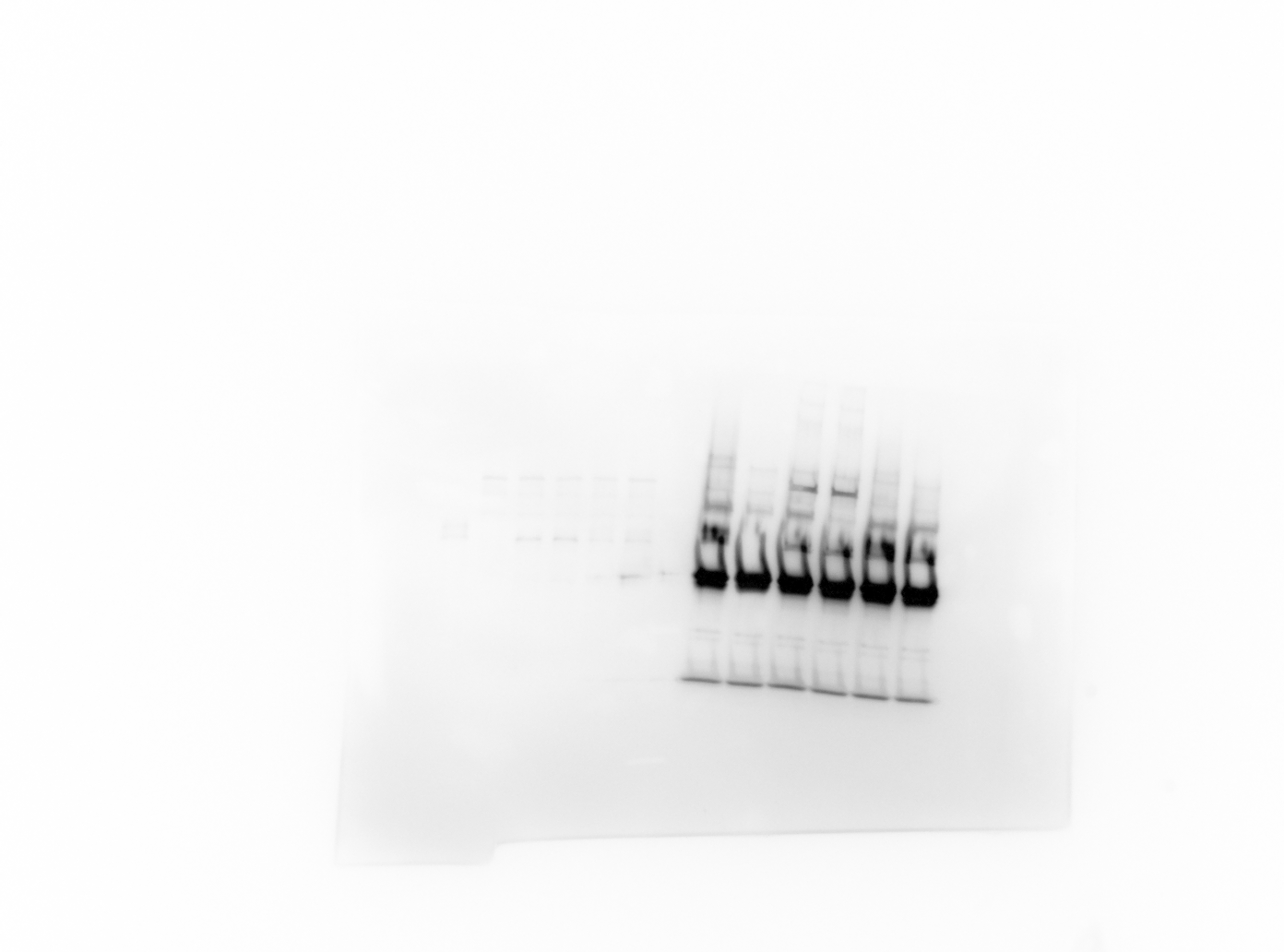

Supplement: Source data 2. [file elife-74531-data2.zip › Source Data Figures/Source Data Figure 3B/Fig3B V5.tif]

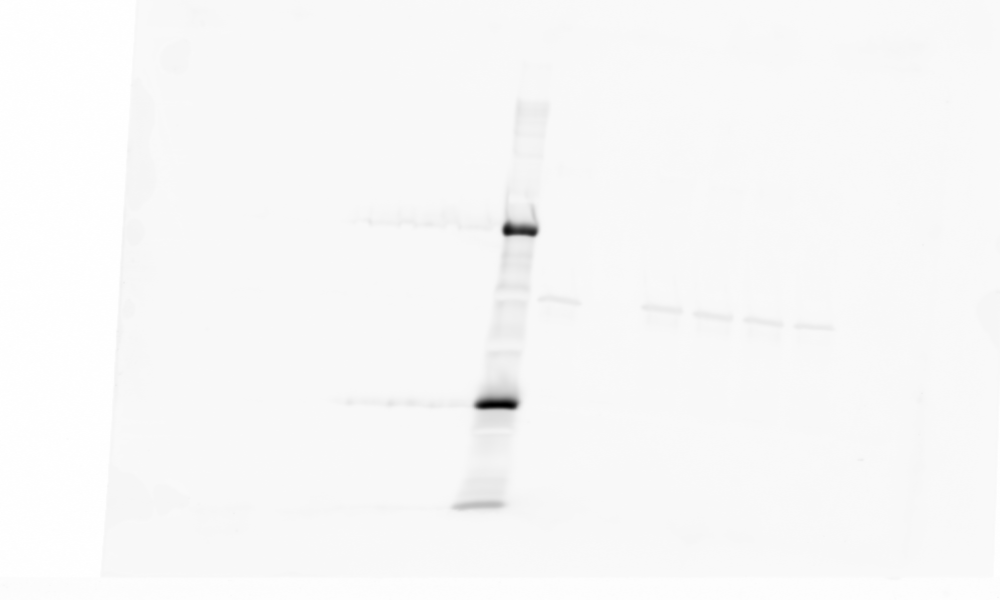

Supplement: Source data 2. [file elife-74531-data2.zip › Source Data Figures/Source Data Figure 3B/Fig3B flag.tif]

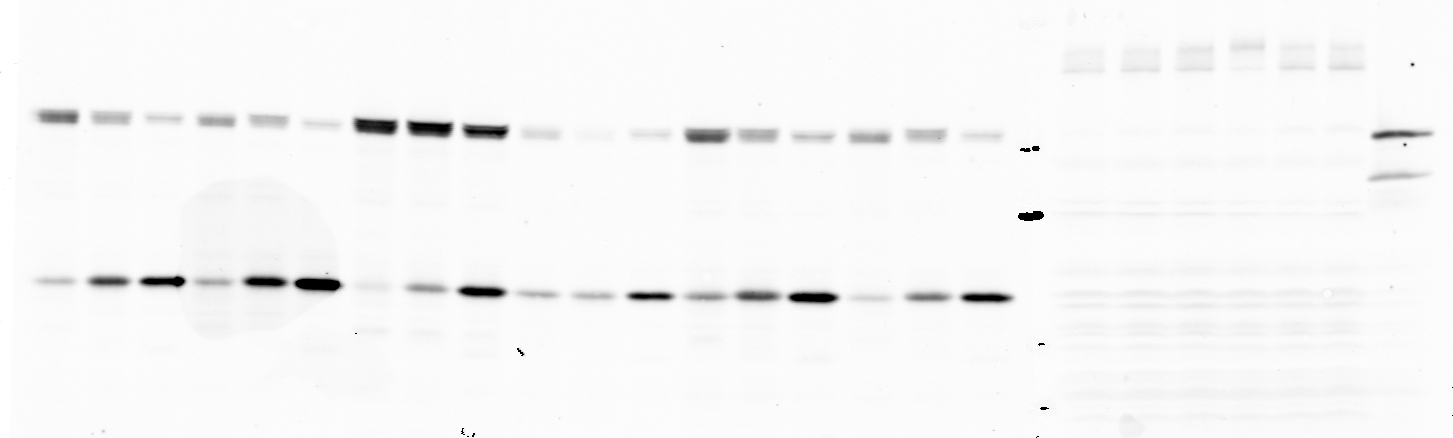

Supplement: Source data 2. [file elife-74531-data2.zip › Source Data Figures/Source Data Figure 1D/Figure 1D GFP.TIF]

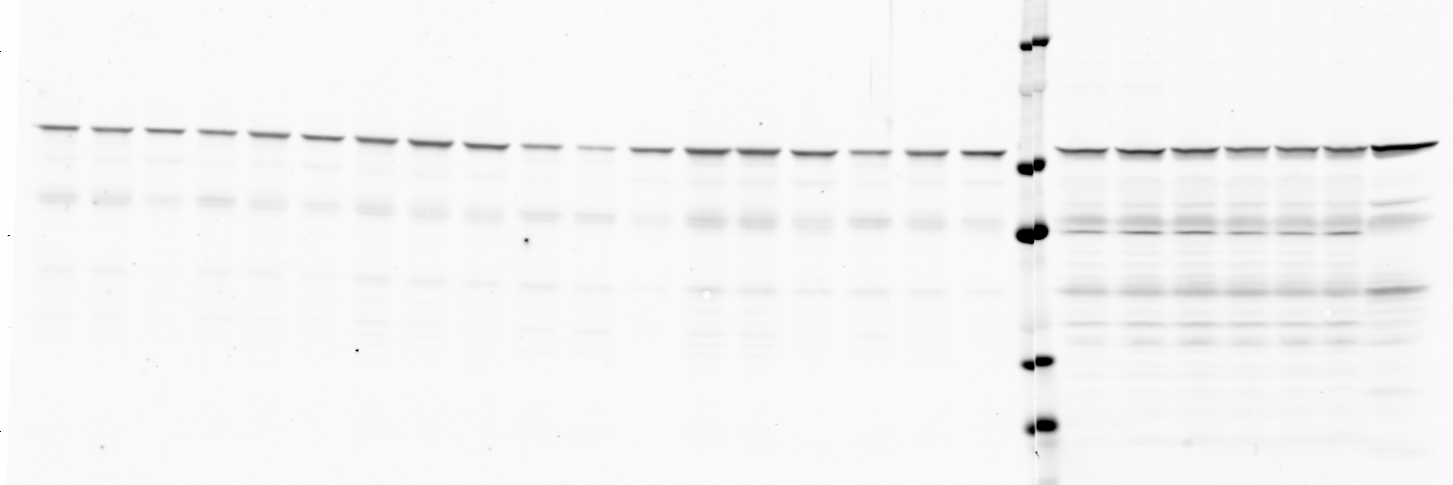

Supplement: Source data 2. [file elife-74531-data2.zip › Source Data Figures/Source Data Figure 1D/Figure 1D HXK.TIF]

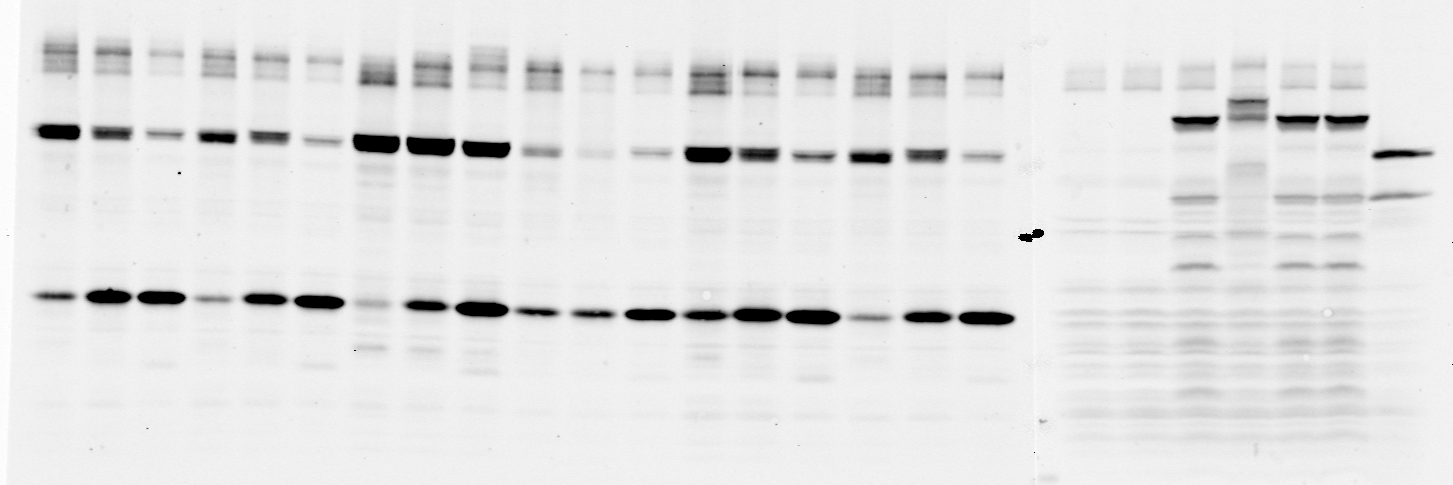

Supplement: Source data 2. [file elife-74531-data2.zip › Source Data Figures/Source Data Figure 1D/Figure 1D MYC.TIF]

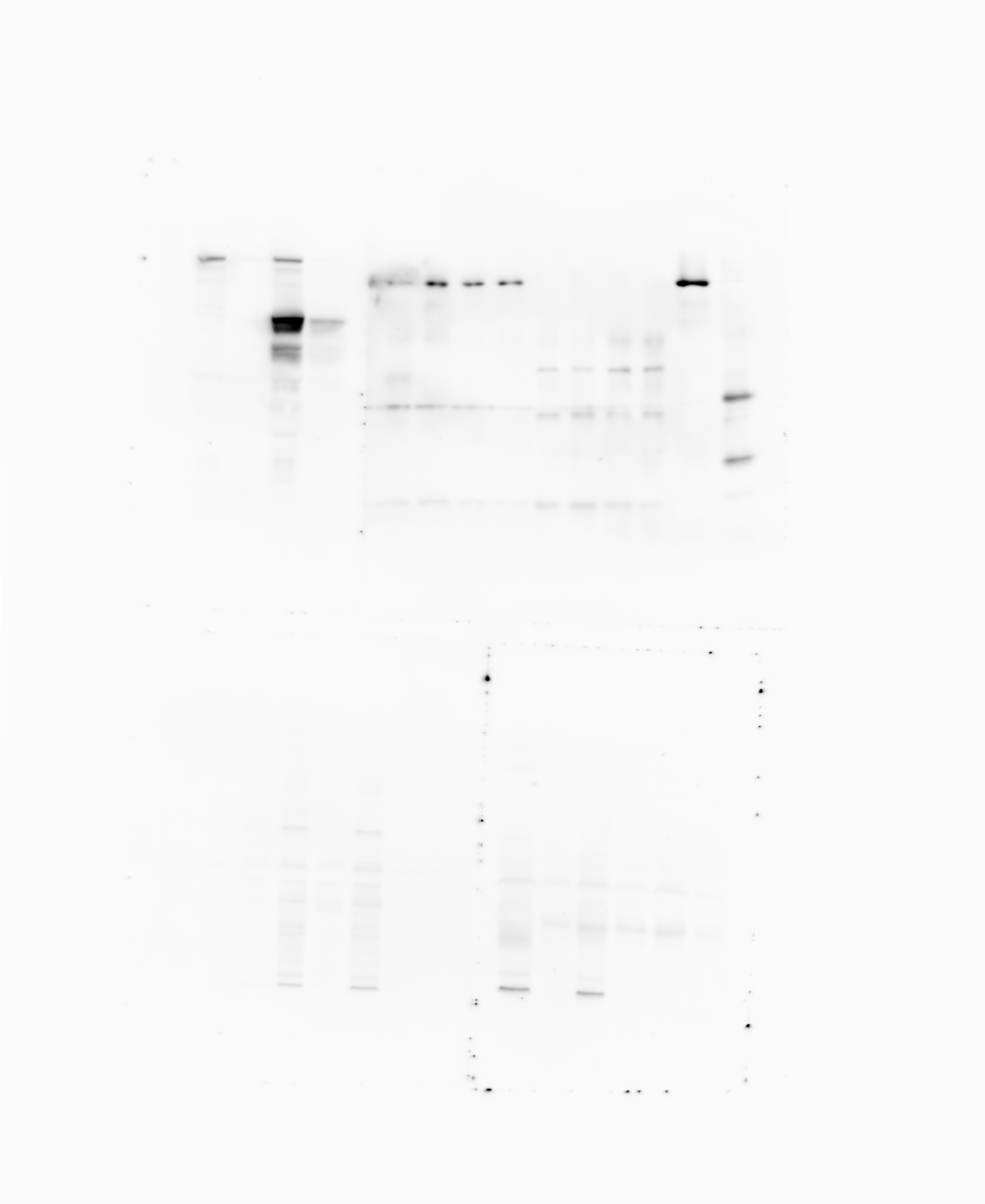

Supplement: Source data 2. [file elife-74531-data2.zip › Source Data Figures/Source Data Figure 1C/Figure 1C V5.tif]

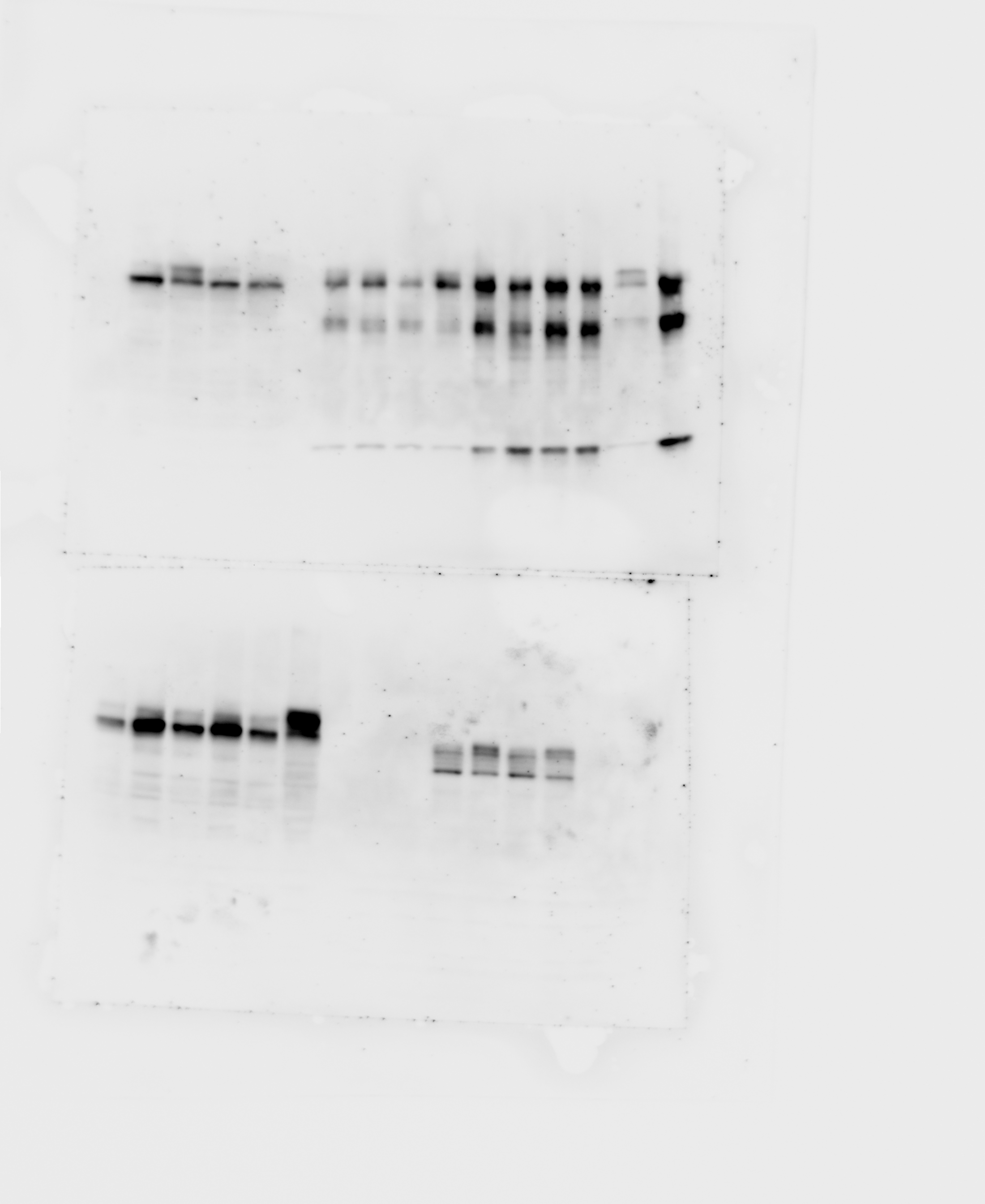

Supplement: Source data 2. [file elife-74531-data2.zip › Source Data Figures/Source Data Figure 1C/Figure 1C MYC.tif]

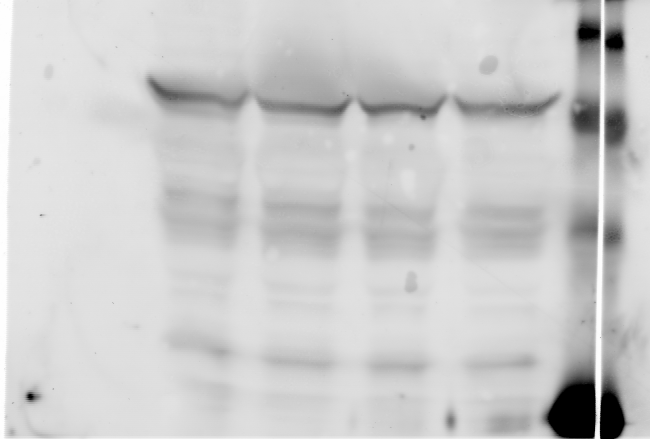

Supplement: Source data 2. [file elife-74531-data2.zip › Source Data Figures/Source Data Figure 1C/Figure 1C HXK.tif]

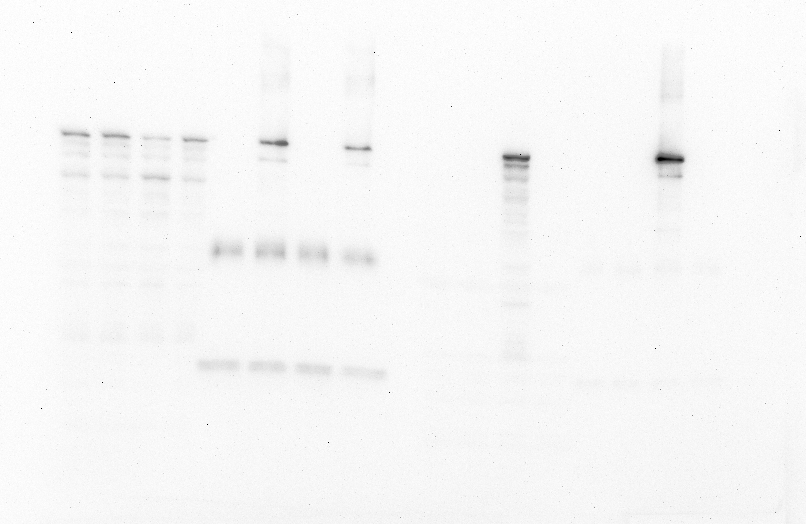

Supplement: Source data 2. [file elife-74531-data2.zip › Source Data Figures/Source Data Figure 2B/Fig2B V5.tif]

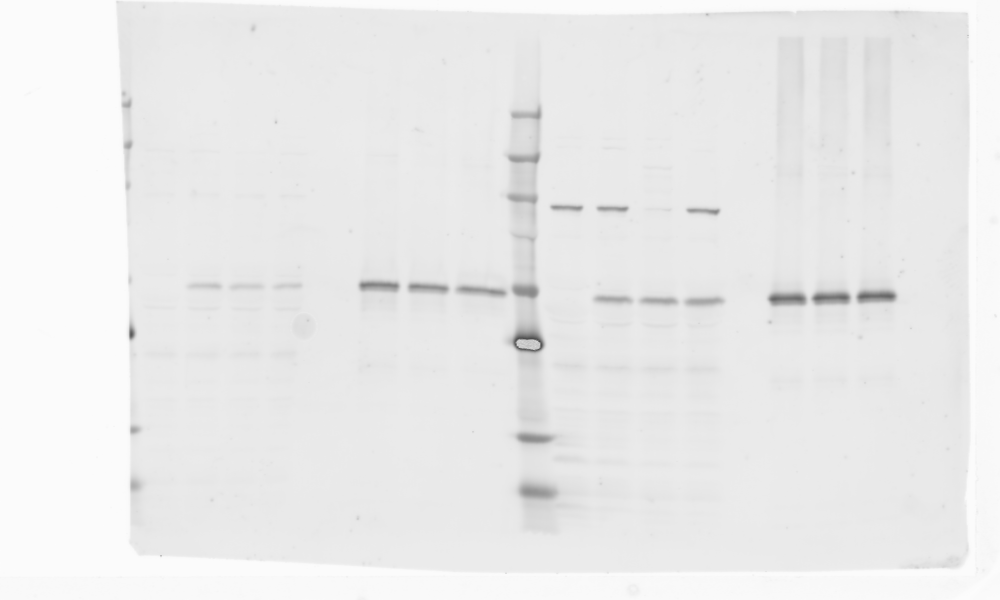

Supplement: Source data 2. [file elife-74531-data2.zip › Source Data Figures/Source Data Figure 2B/Fig2B FLAG.tiff]

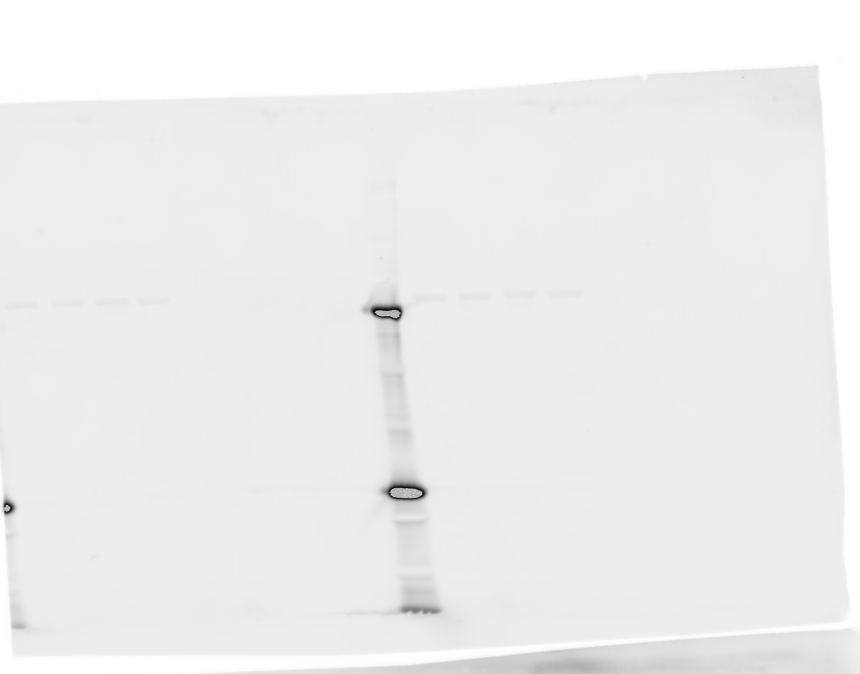

Supplement: Source data 2. [file elife-74531-data2.zip › Source Data Figures/Source Data Figure 2B/Fig2B HSC82.tif]

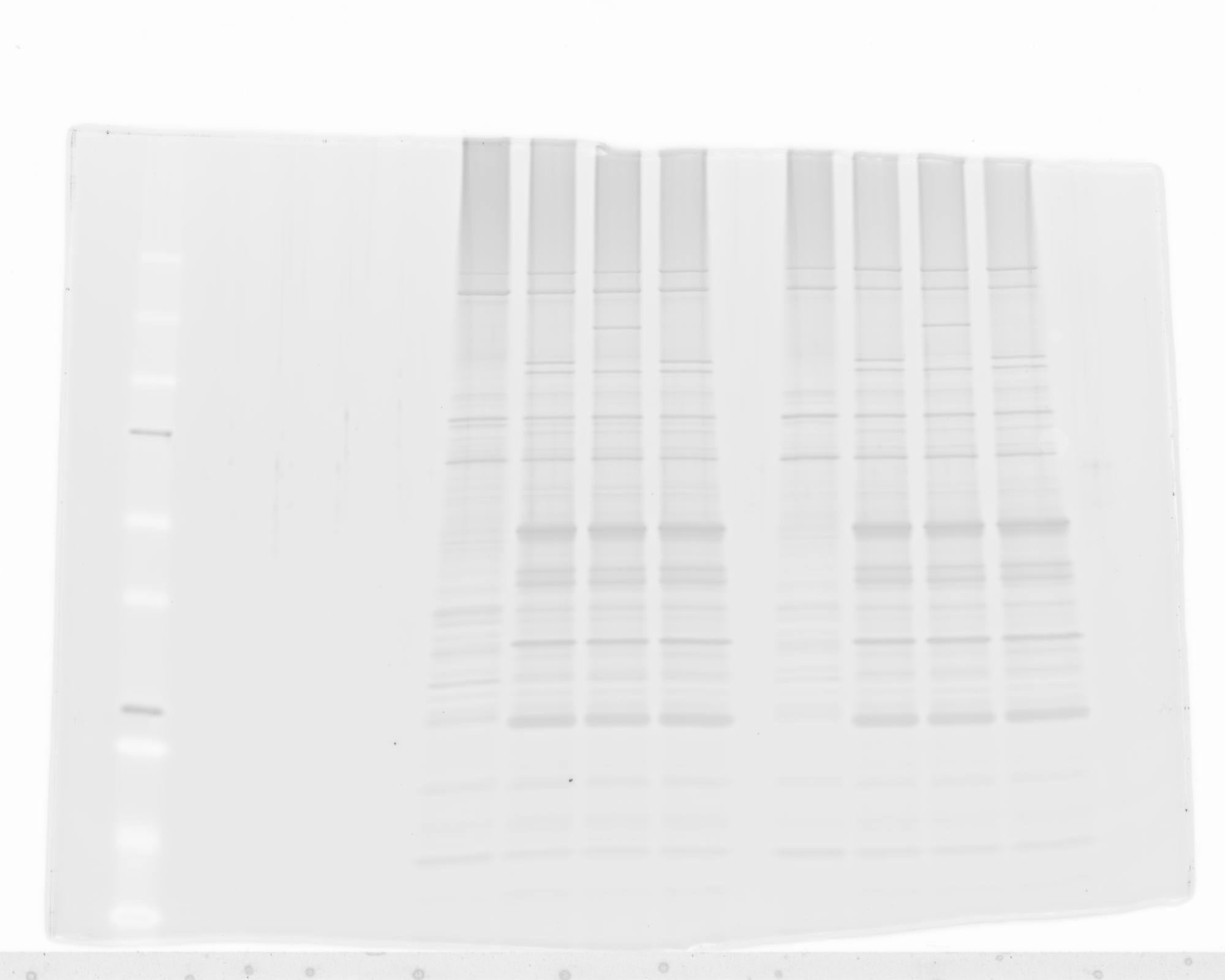

Supplement: Source data 2. [file elife-74531-data2.zip › Source Data Figures/Source Data Figure 2B/Fig2B sypro.tif]

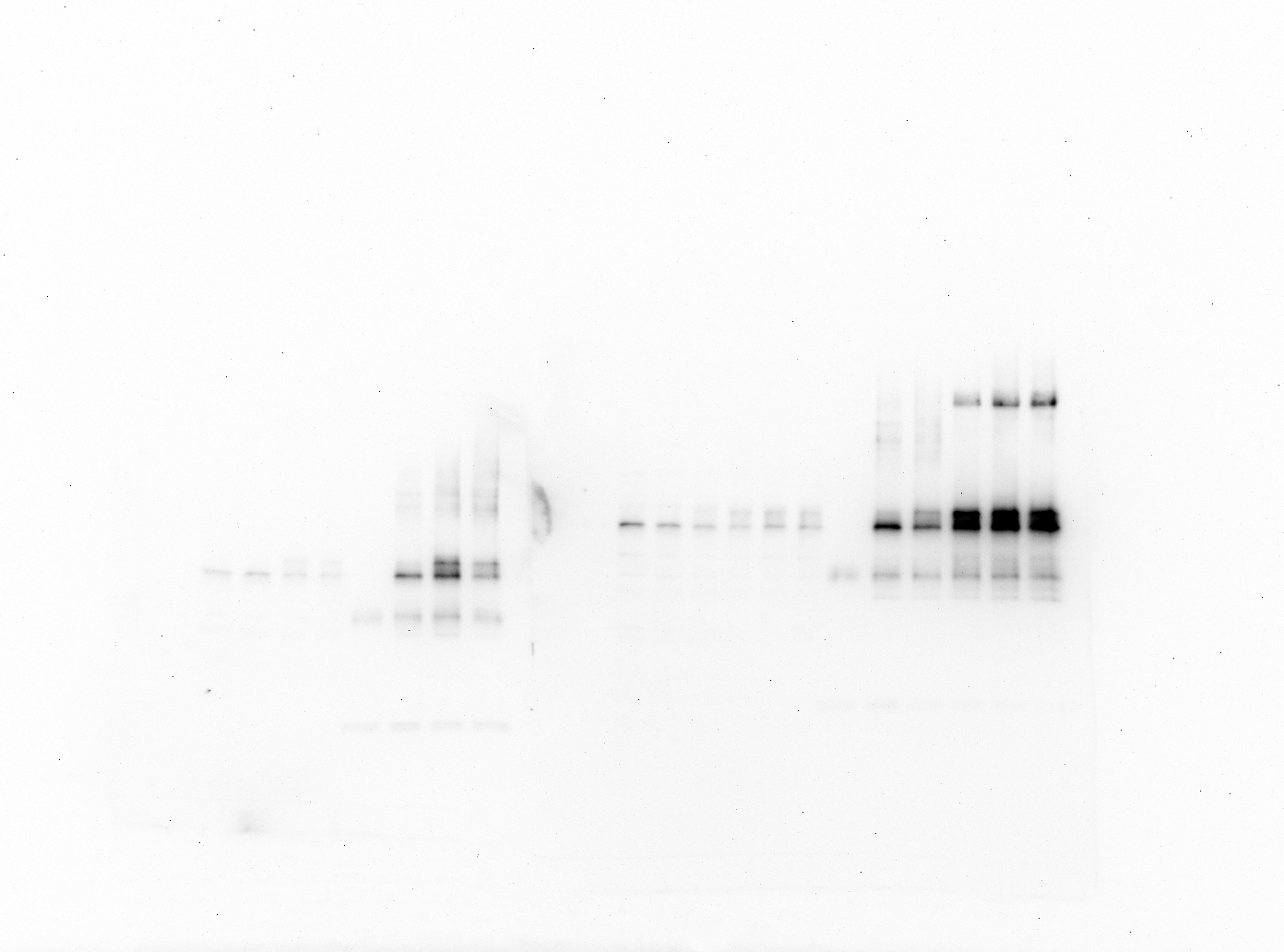

Supplement: Source data 2. [file elife-74531-data2.zip › Source Data Figures/Source Data Figure 2C/Fig2C MYC.tif]

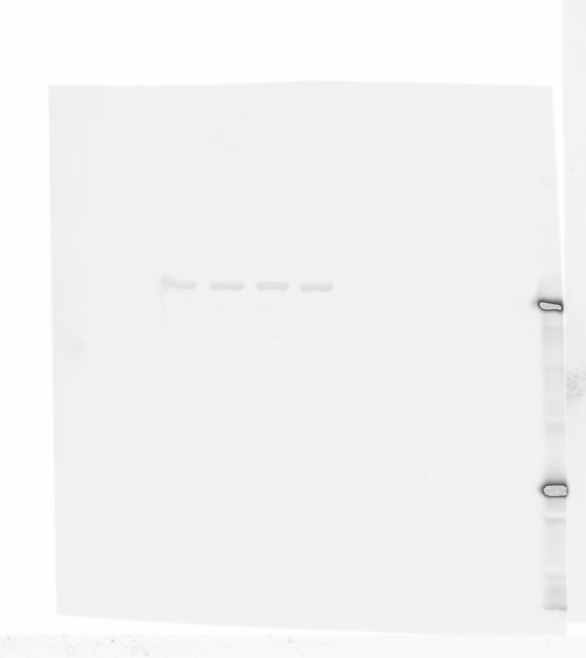

Supplement: Source data 2. [file elife-74531-data2.zip › Source Data Figures/Source Data Figure 2C/Fig2C HSC82.tif]

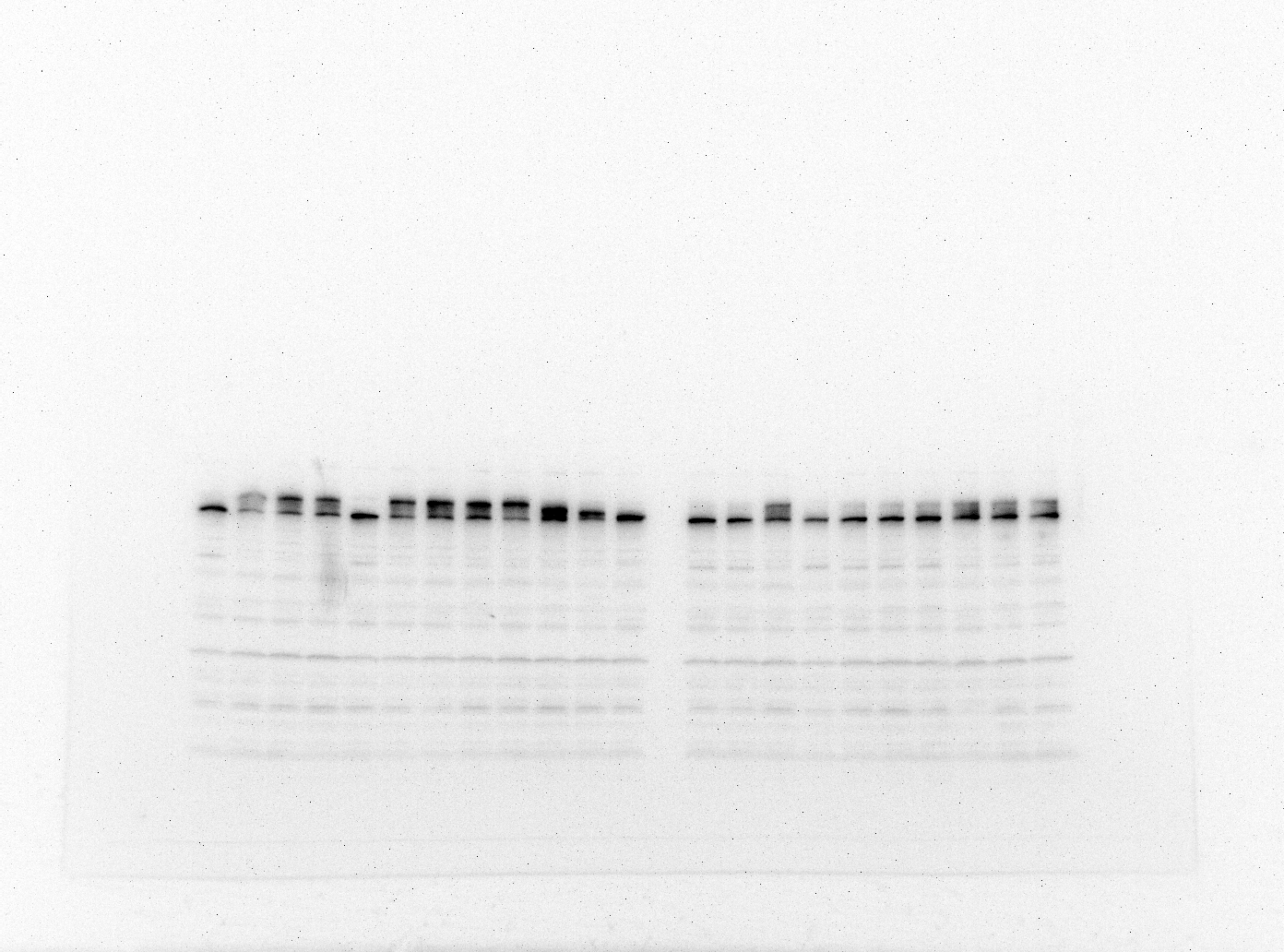

Supplement: Source data 2. [file elife-74531-data2.zip › Source Data Figures/Source Data Figure 1B/Fig 1B MYC.tif]

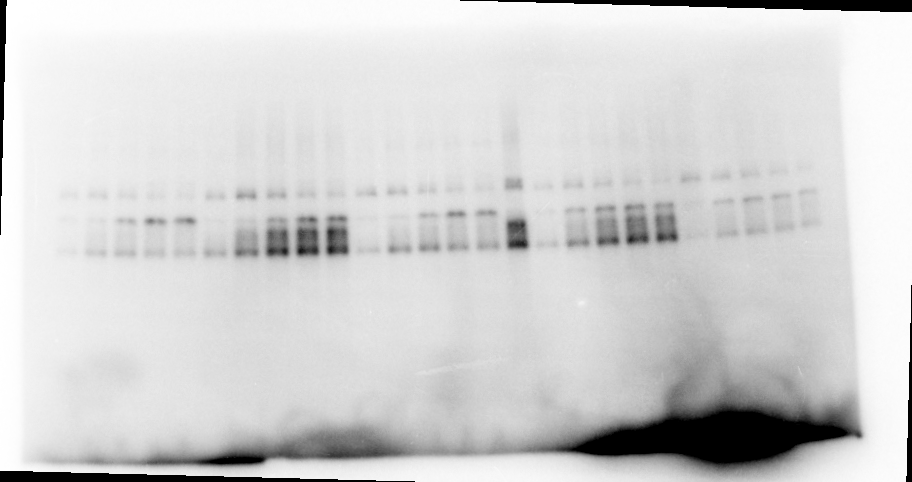

Supplement: Source data 2. [file elife-74531-data2.zip › Source Data Figures/Source Data Figure 3G/Figure 3G.tif]

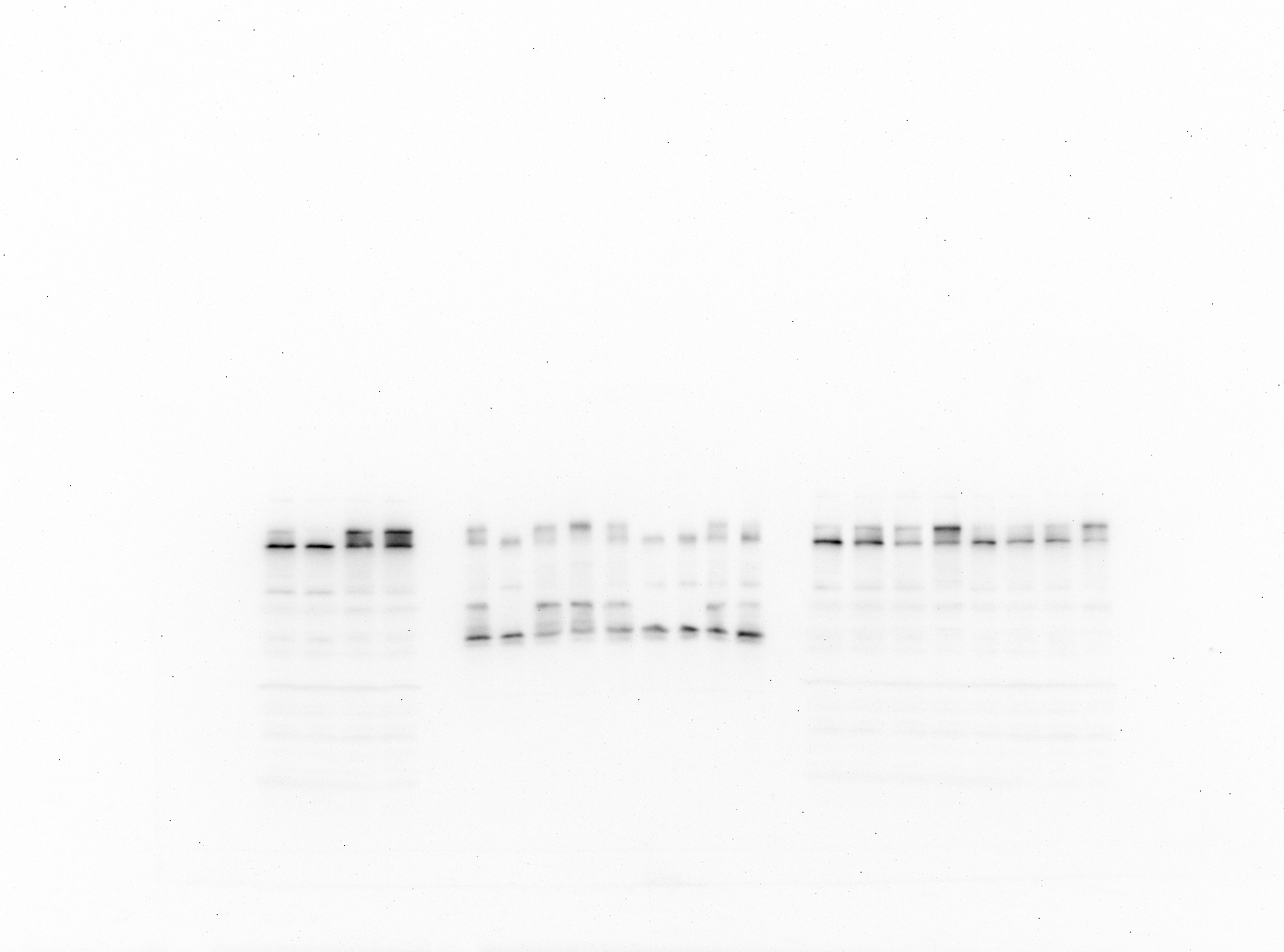

Supplement: Source data 2. [file elife-74531-data2.zip › Source Data Figures/Source Data Figure 3A/Fig3A MYC.tif]

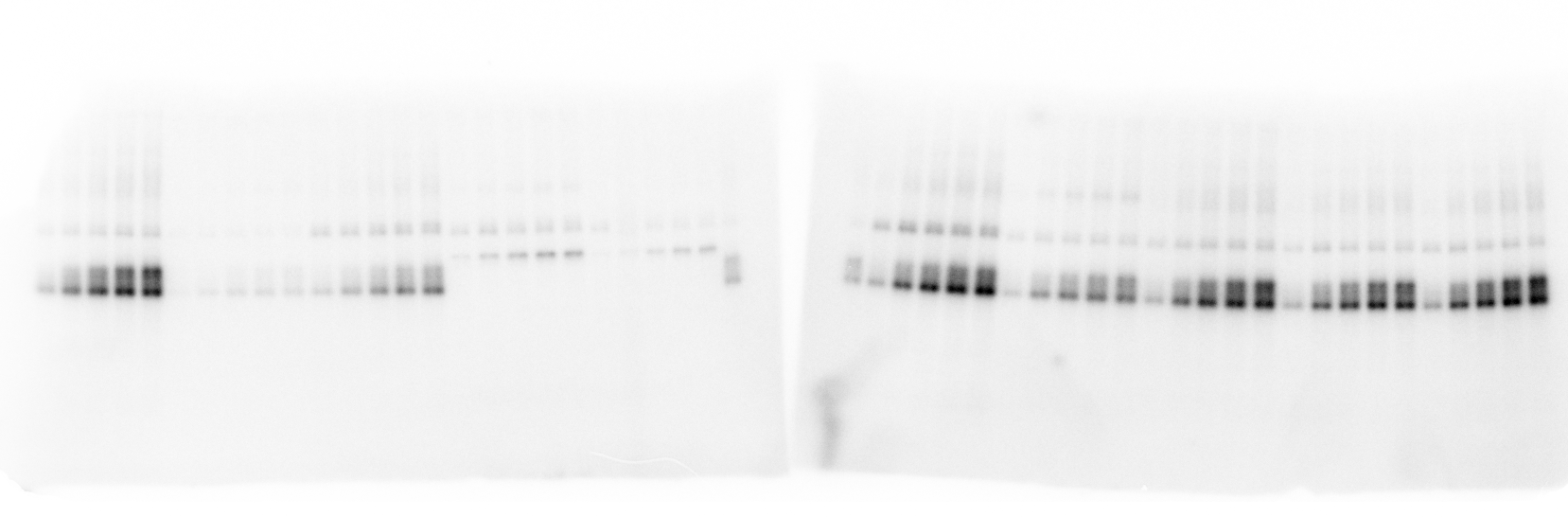

Supplement: Source data 2. [file elife-74531-data2.zip › Source Data Figures/Source Data Figure 3F/Figure 3F.tif]

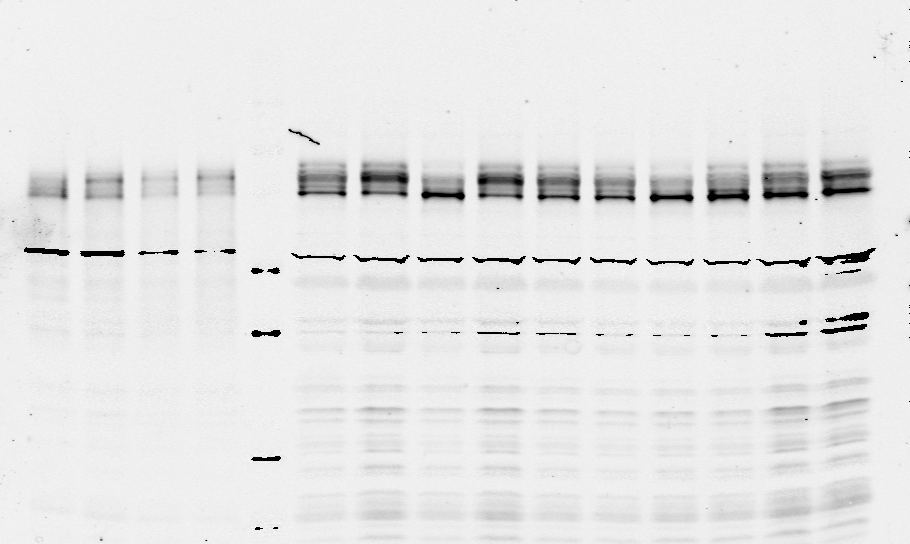

Supplement: Source data 3. [file elife-74531-data3.zip › Source Data - Figure supplements/Source Data Figure 3-figure supplement 1B/Fig 3-FS1B MYC.TIF]

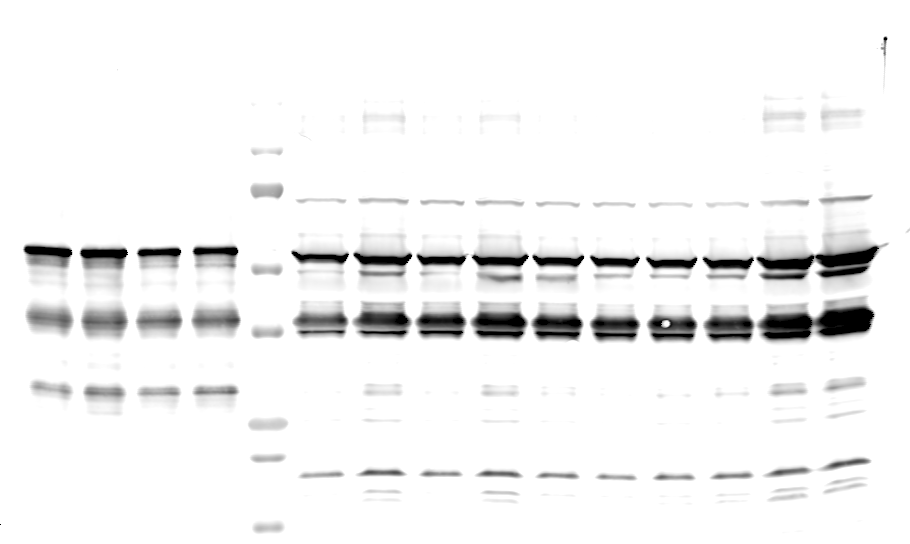

Supplement: Source data 3. [file elife-74531-data3.zip › Source Data - Figure supplements/Source Data Figure 3-figure supplement 1B/Fig 3-FS1B HXK.TIF]

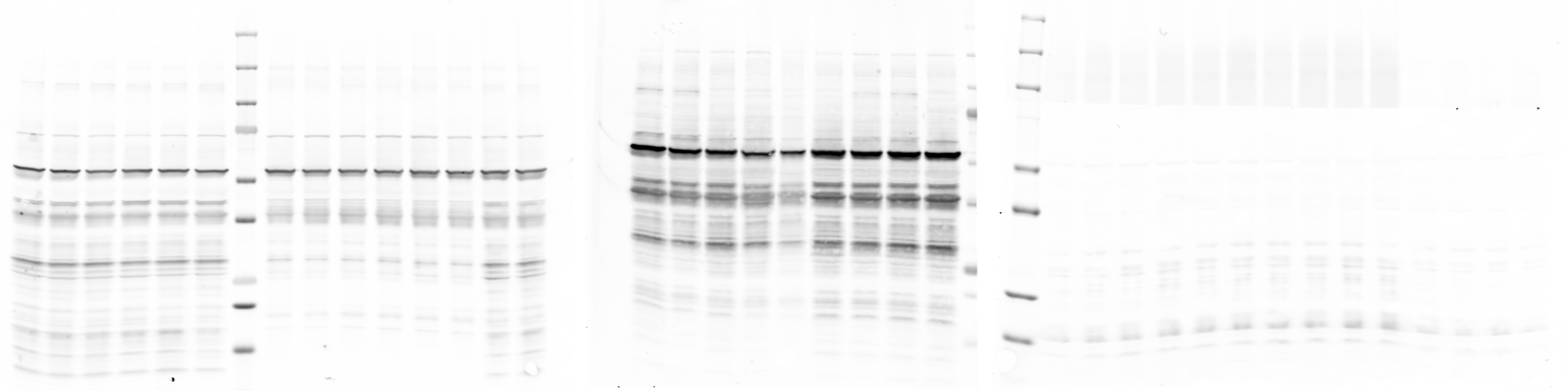

Supplement: Source data 3. [file elife-74531-data3.zip › Source Data - Figure supplements/Source Data Figure 3-figure supplement 1E/Fig 3-FS1E HXK.TIF]

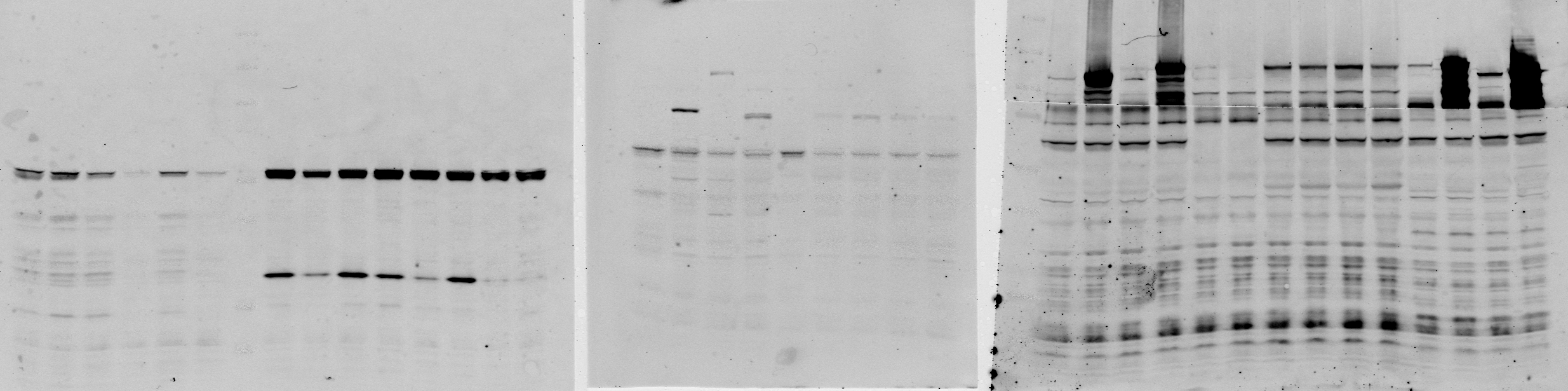

Supplement: Source data 3. [file elife-74531-data3.zip › Source Data - Figure supplements/Source Data Figure 3-figure supplement 1E/Fig 3-FS1E GFP.TIF]

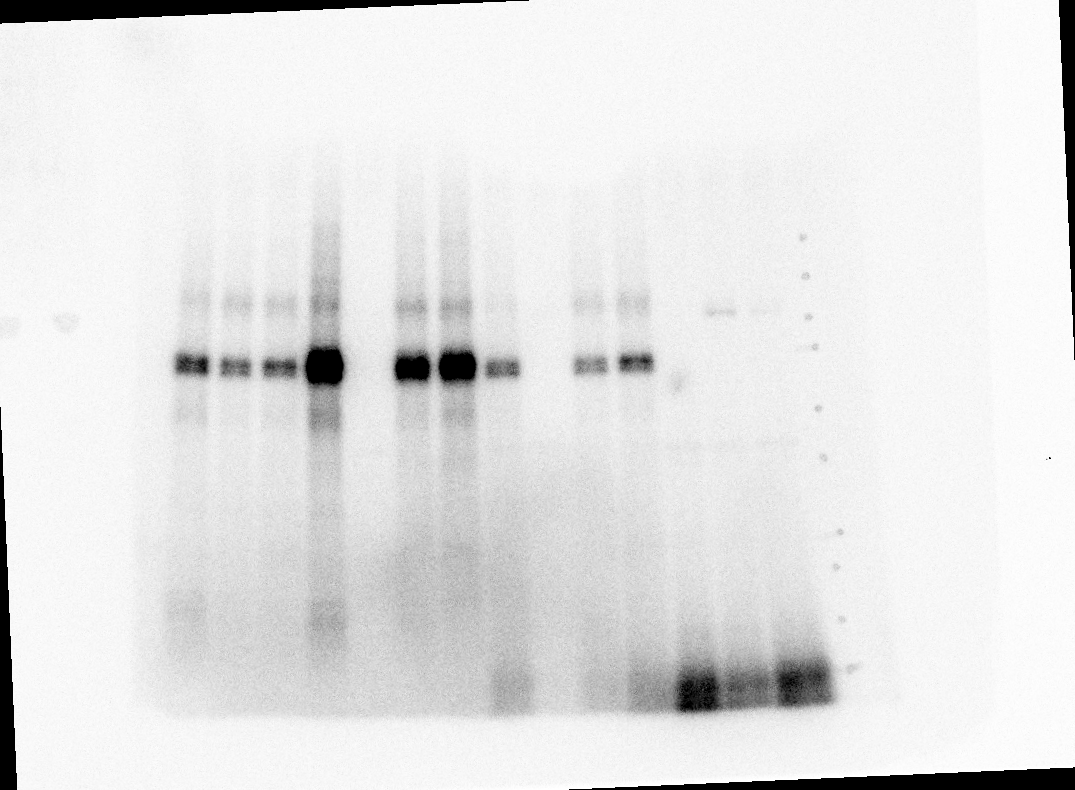

Supplement: Source data 3. [file elife-74531-data3.zip › Source Data - Figure supplements/Source Data Figure 3-figure supplement 2C/Fig 3-FS2C.tif]

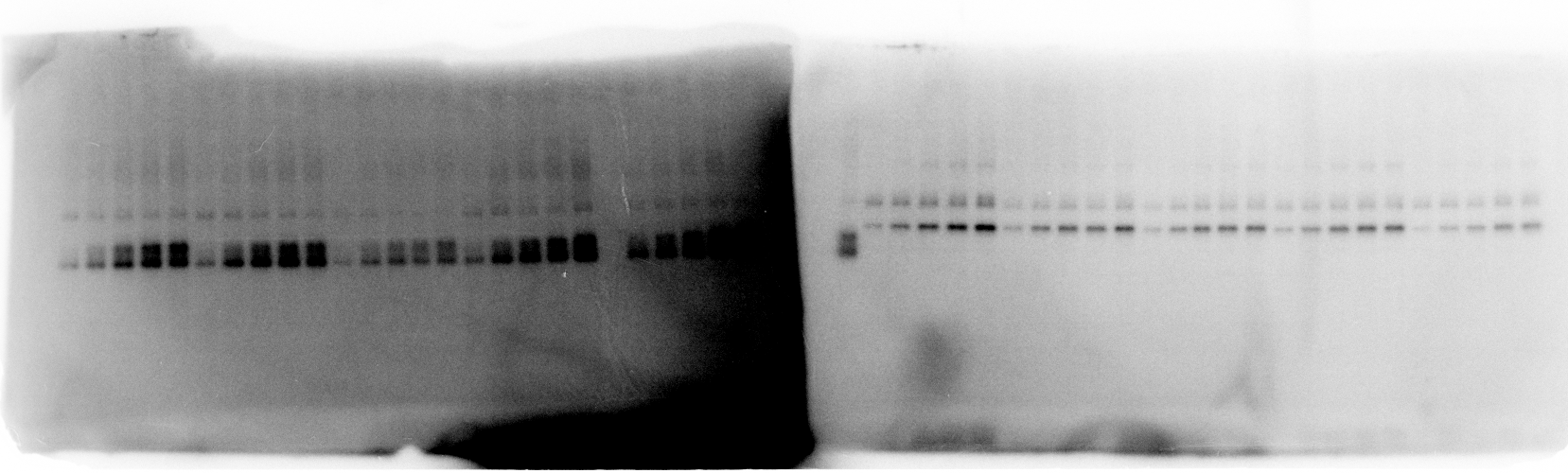

Supplement: Source data 3. [file elife-74531-data3.zip › Source Data - Figure supplements/Source Data Figure 3-figure supplement 2E/Fig 3-FS2E.tif]

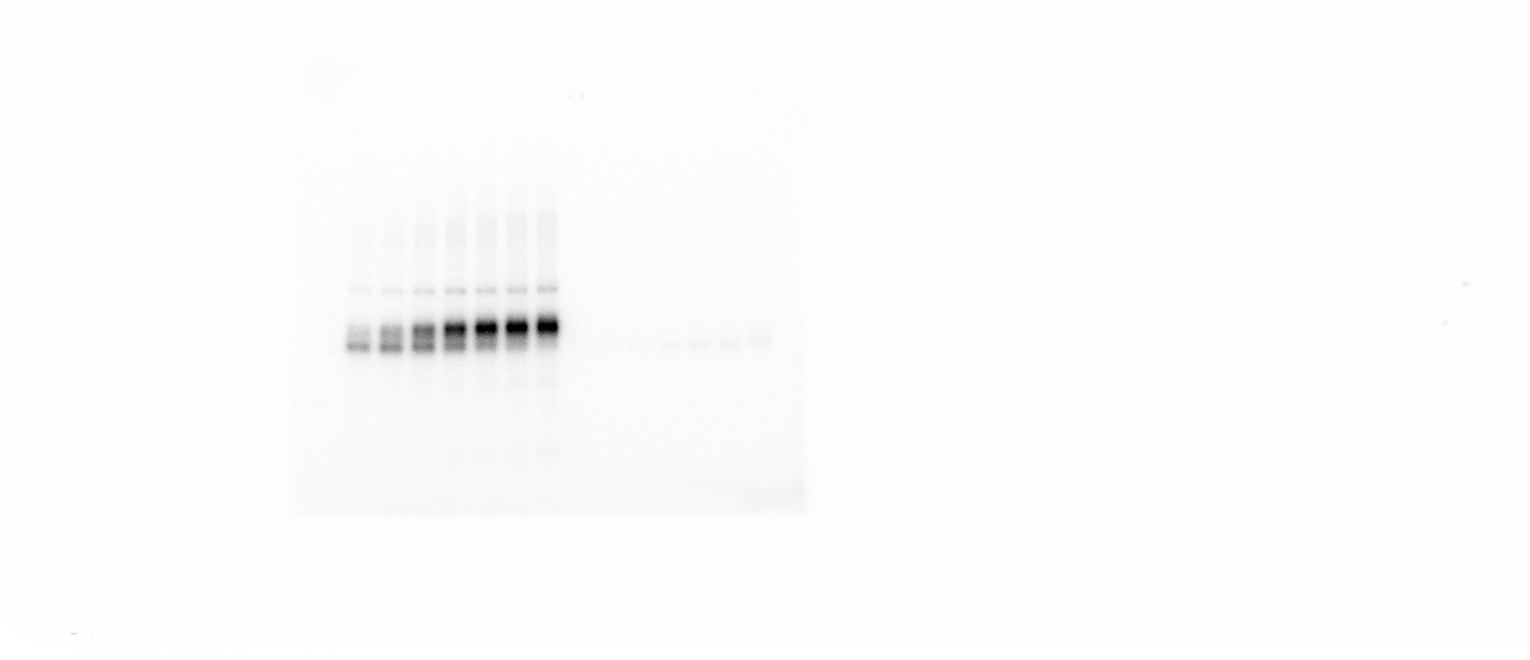

Supplement: Source data 3. [file elife-74531-data3.zip › Source Data - Figure supplements/Source Data Figure 3-figure supplement 2B/Fig 3-FS2B.tif]

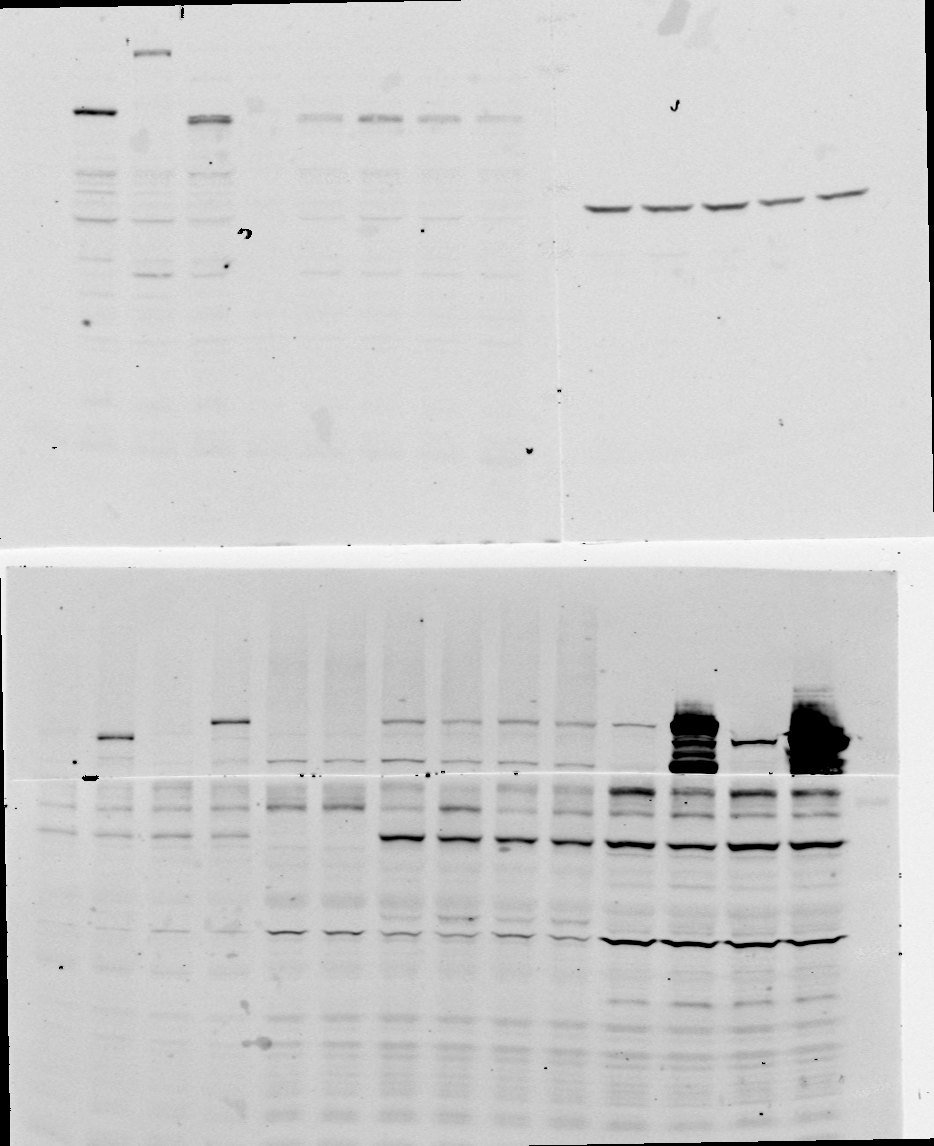

Supplement: Source data 3. [file elife-74531-data3.zip › Source Data - Figure supplements/Source Data Figure 3-figure supplement 1C/Fig 3-FS1C mCherry.TIF]

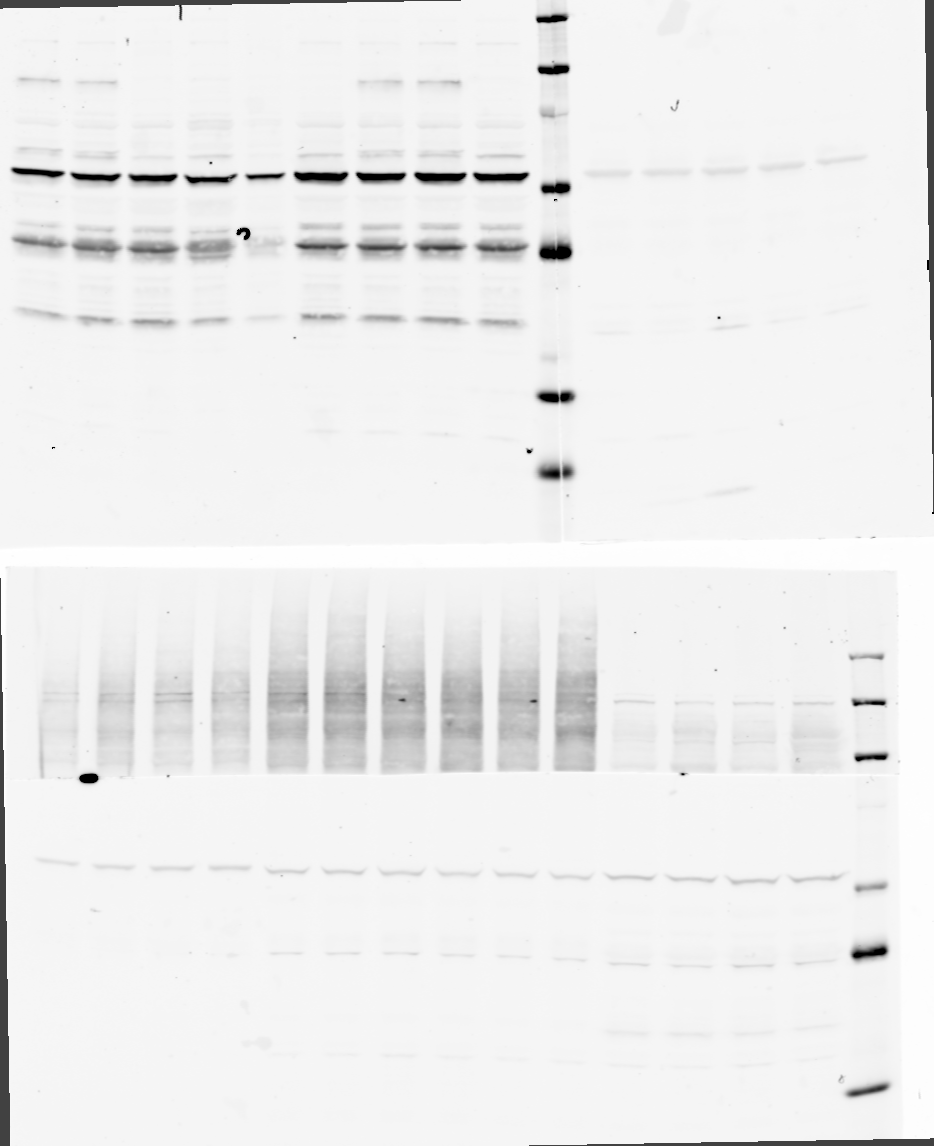

Supplement: Source data 3. [file elife-74531-data3.zip › Source Data - Figure supplements/Source Data Figure 3-figure supplement 1C/Fig 3-FS1C HXK.TIF]

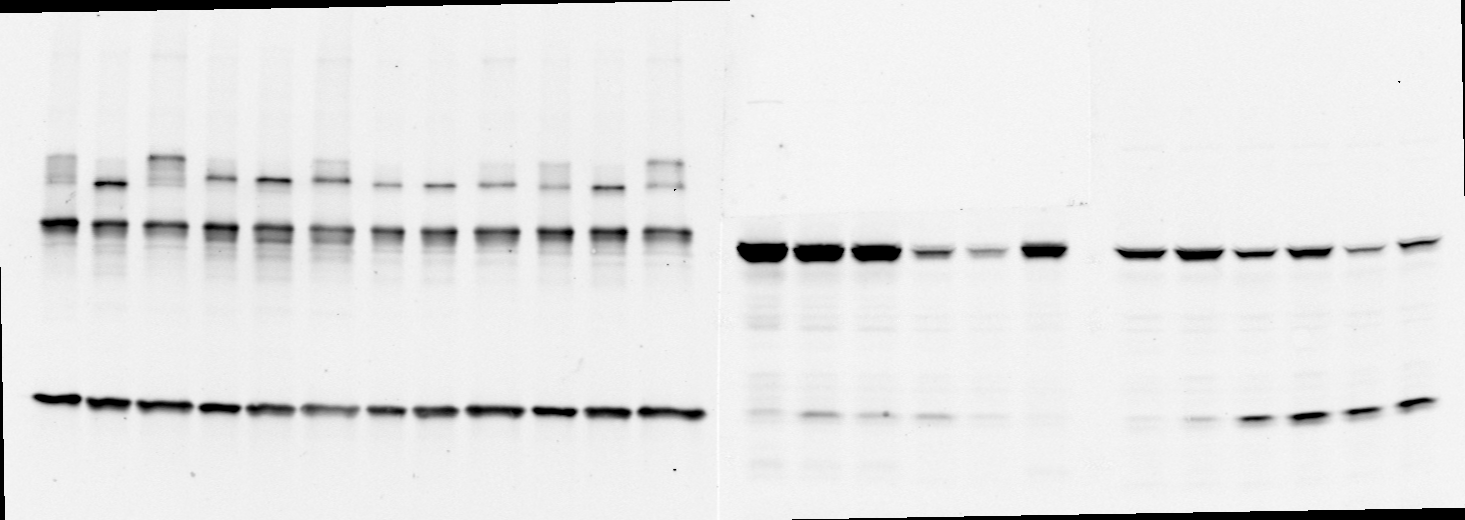

Supplement: Source data 3. [file elife-74531-data3.zip › Source Data - Figure supplements/Source Data Figure 4-figure supplement 1F/Fig 4-FS1F MYC.TIF]

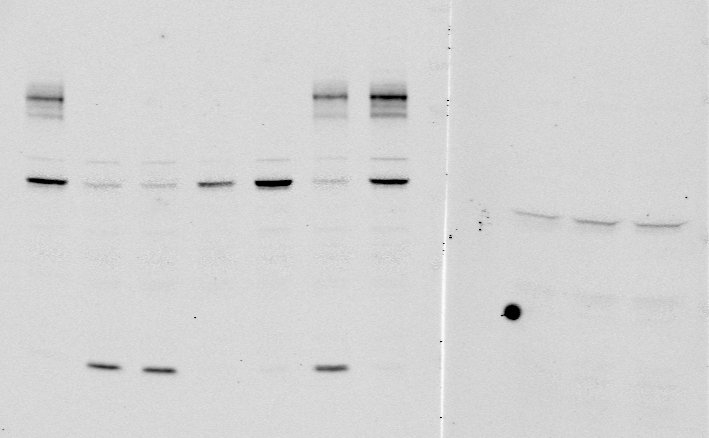

Supplement: Source data 3. [file elife-74531-data3.zip › Source Data - Figure supplements/Source Data Figure 1-figure supplement 1A/Fig 1-FS1A GFP.TIF]

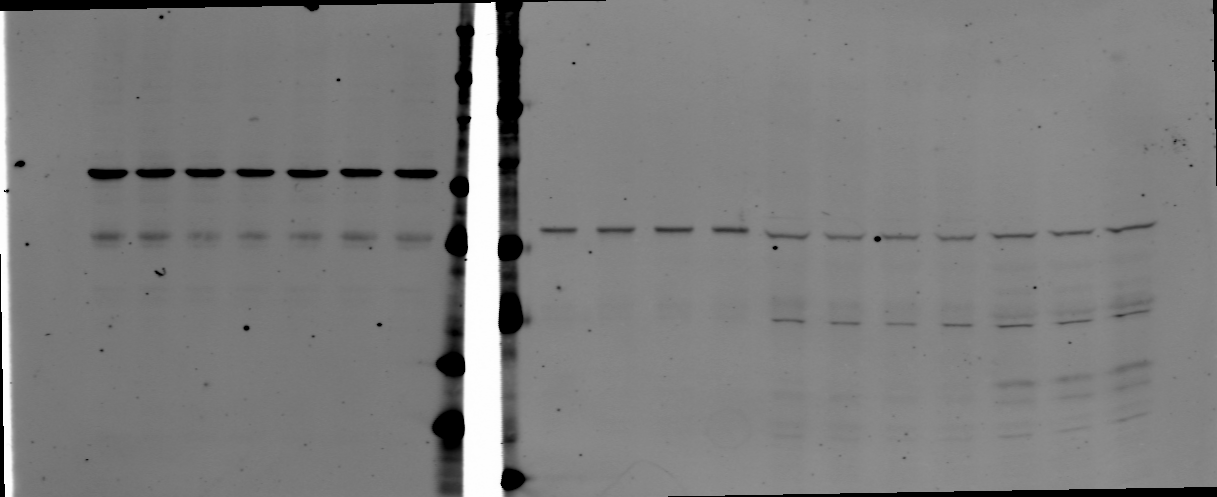

Supplement: Source data 3. [file elife-74531-data3.zip › Source Data - Figure supplements/Source Data Figure 1-figure supplement 1A/Fig 1-FS1A HXK.TIF]

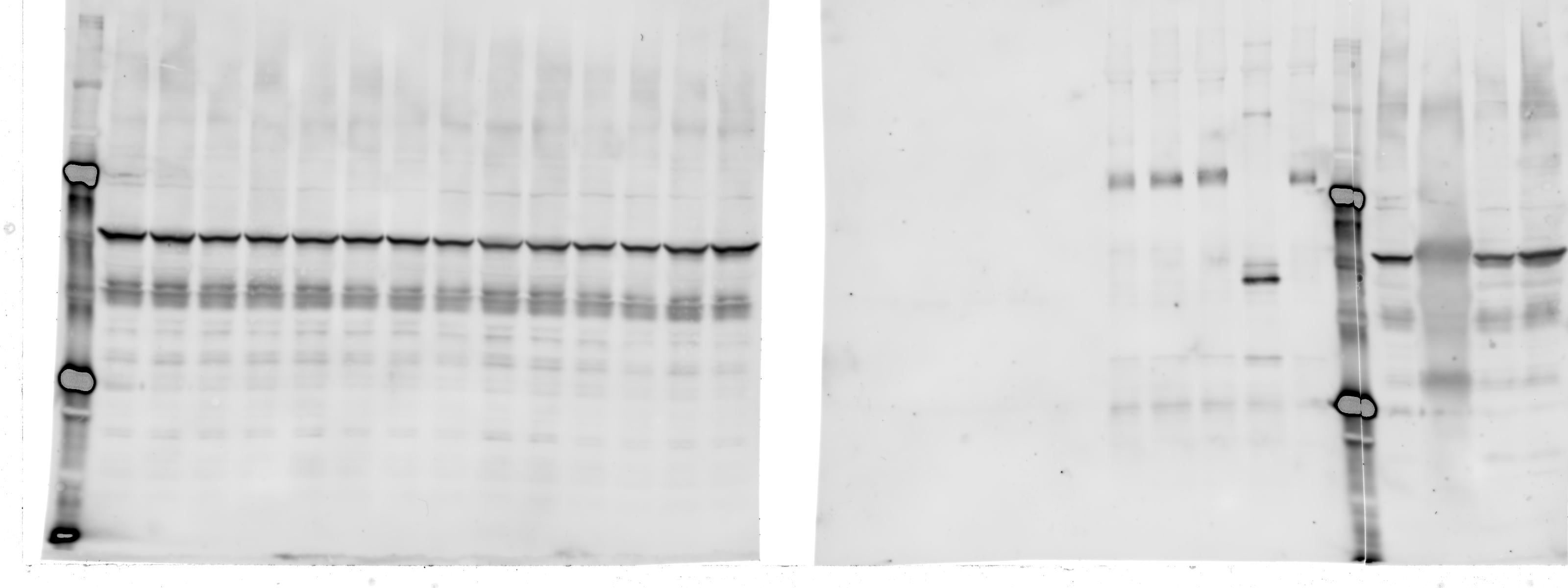

Supplement: Source data 3. [file elife-74531-data3.zip › Source Data - Figure supplements/Source Data Figure 3-figure supplement 1F/Fig 3-FS1F HXK.tif]

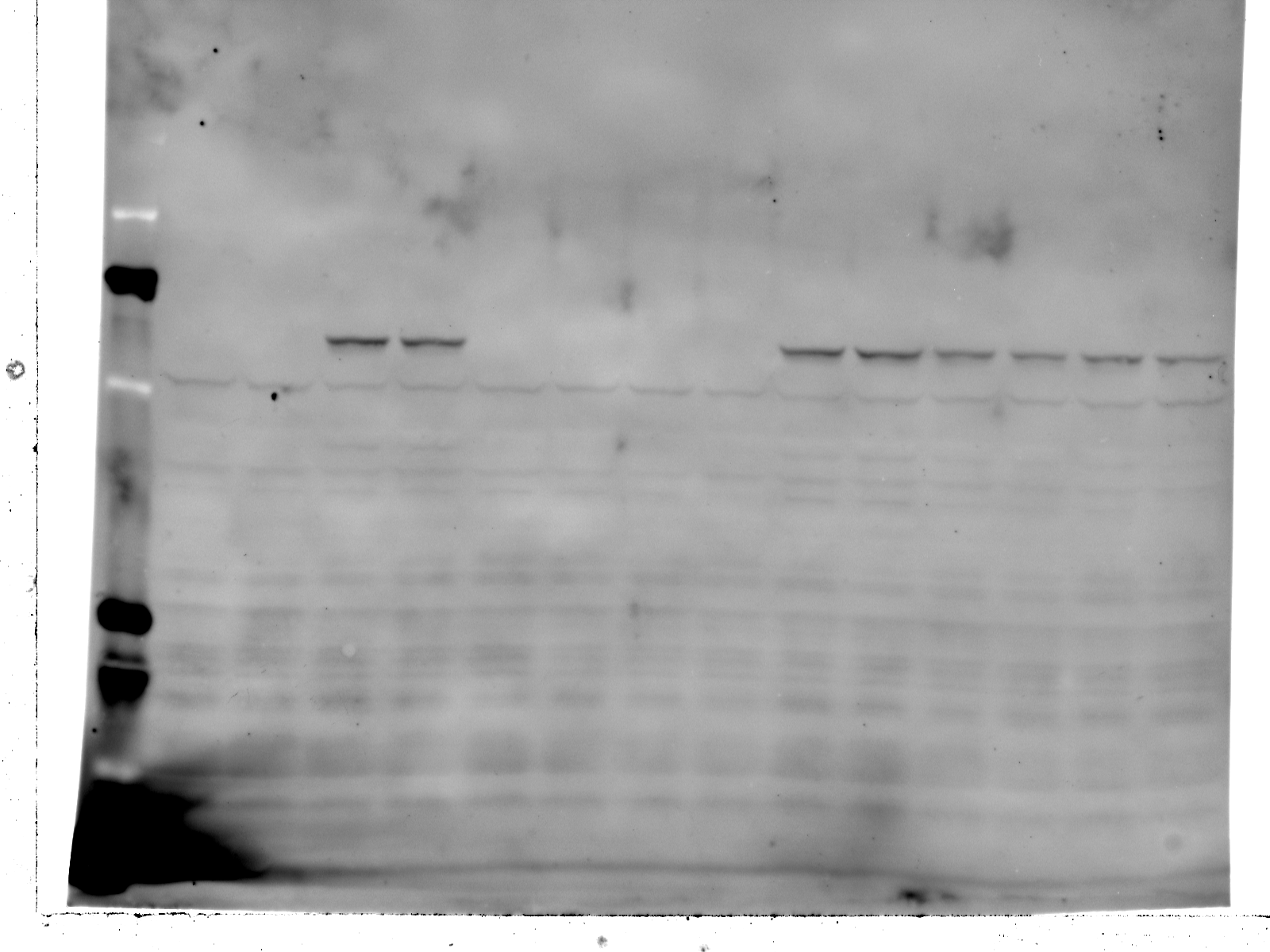

Supplement: Source data 3. [file elife-74531-data3.zip › Source Data - Figure supplements/Source Data Figure 3-figure supplement 1F/Fig 3-FS1F HA.tif]

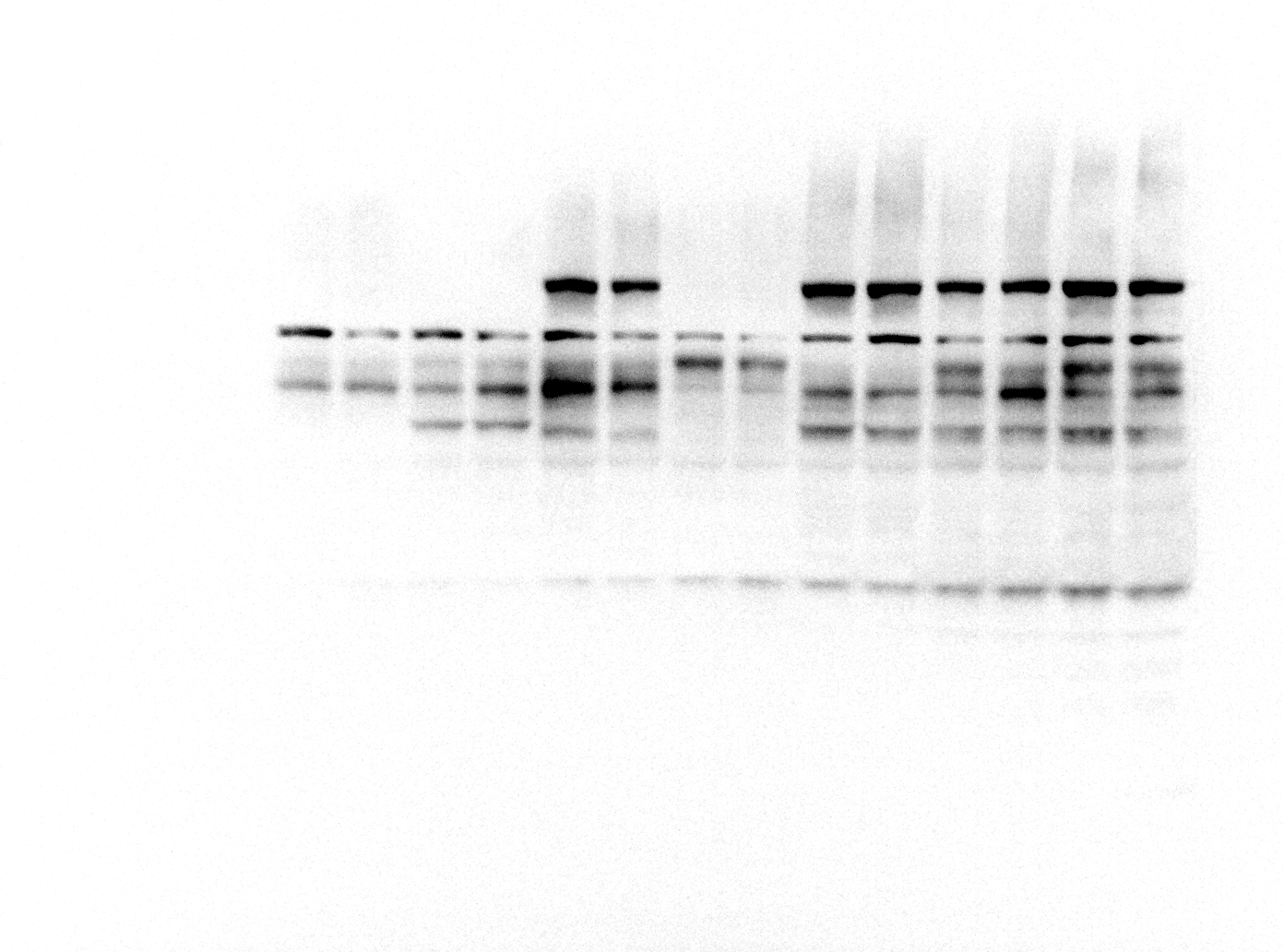

Supplement: Source data 3. [file elife-74531-data3.zip › Source Data - Figure supplements/Source Data Figure 3-figure supplement 1F/Fig 3-FS1F MYC & FLAG.tif]

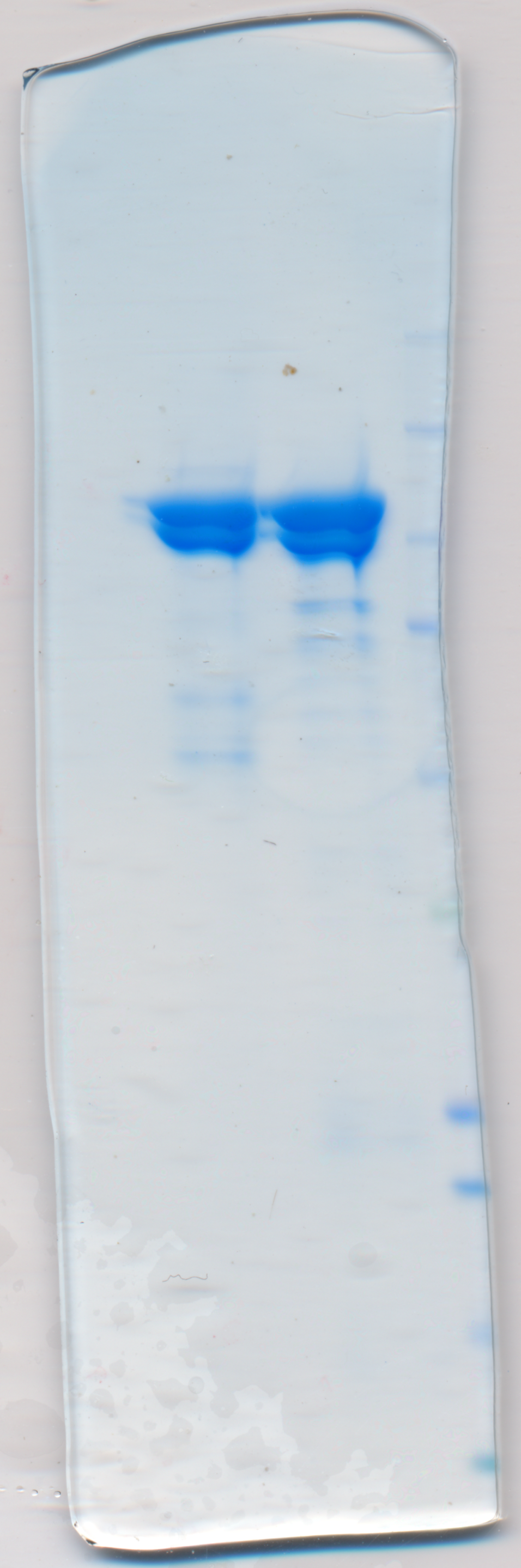

Supplement: Source data 3. [file elife-74531-data3.zip › Source Data - Figure supplements/Source Data Figure 3-figure supplement 2A/Fig 3-FS2A Pex1:6.tif]

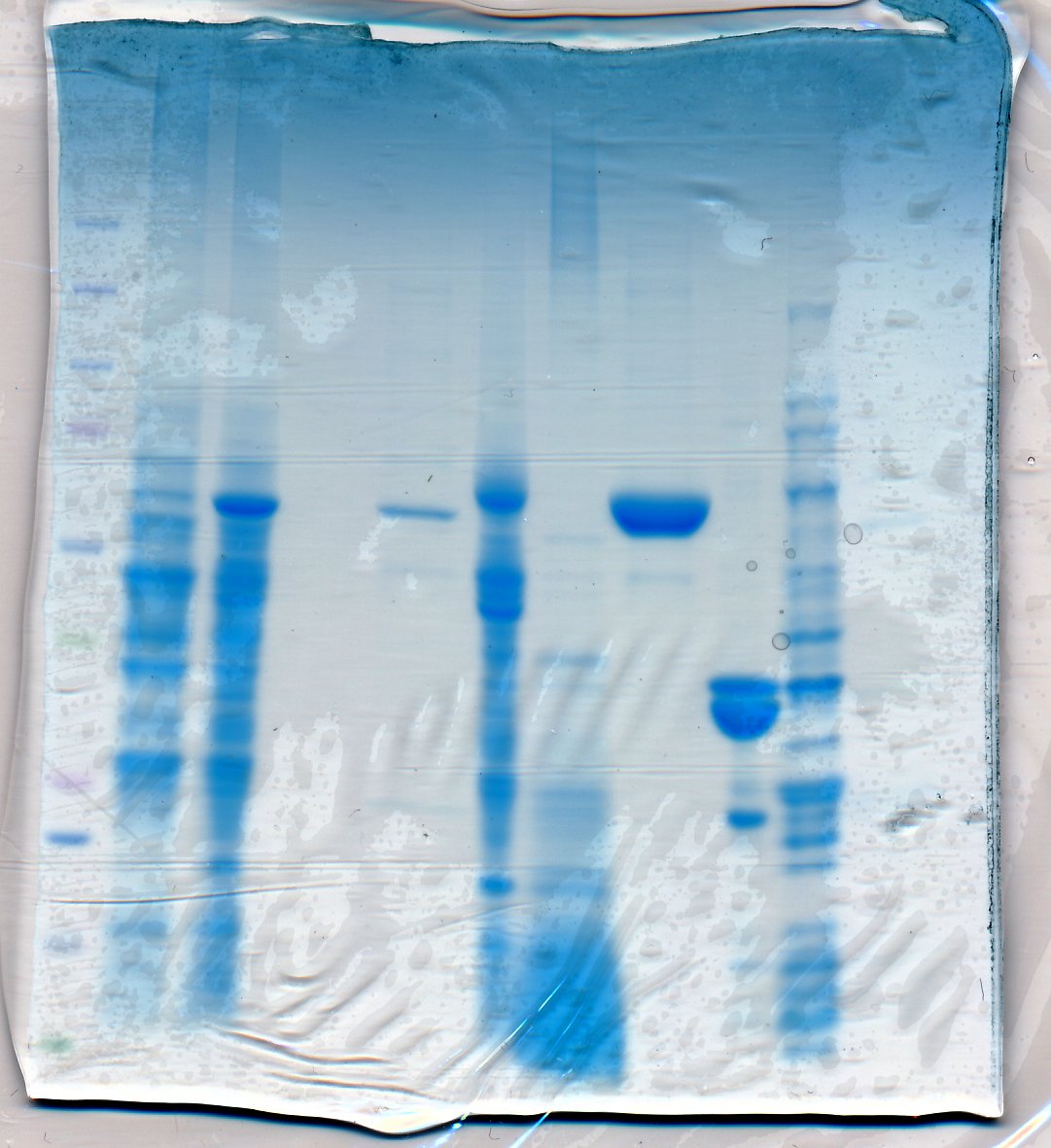

Supplement: Source data 3. [file elife-74531-data3.zip › Source Data - Figure supplements/Source Data Figure 3-figure supplement 2A/Fig 3-FS2A Hrr25.jpg]

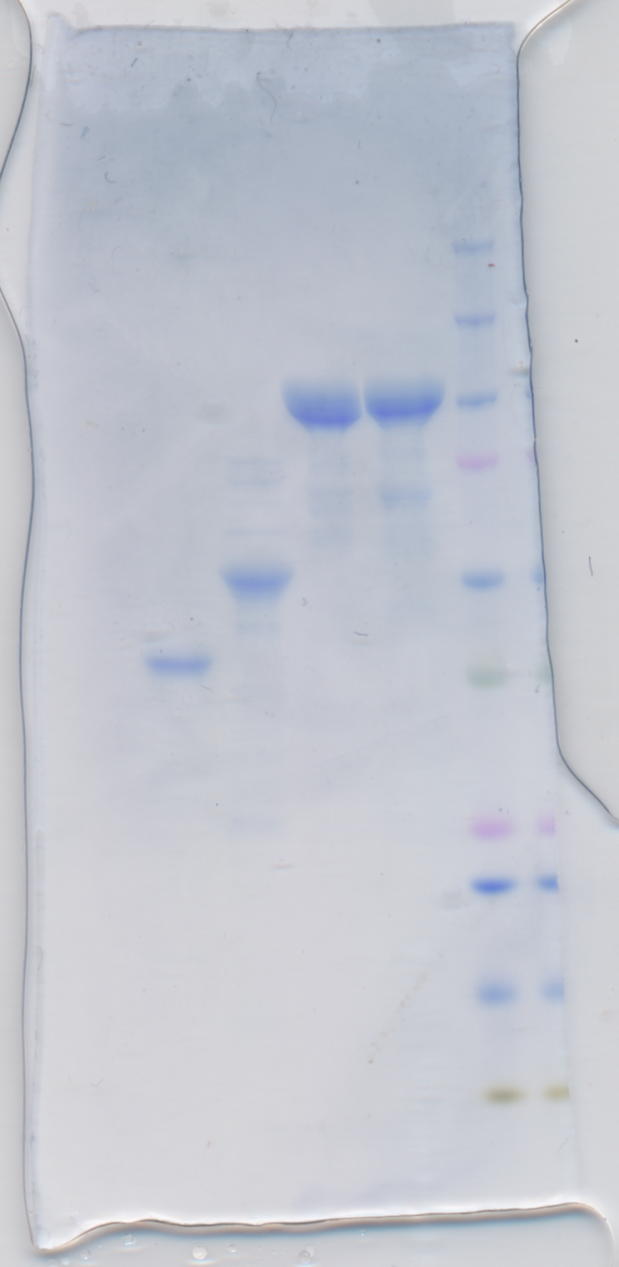

Supplement: Source data 3. [file elife-74531-data3.zip › Source Data - Figure supplements/Source Data Figure 3-figure supplement 2A/Fig 3-FS2A Cdc48, Atg19 and Atg36.tif]

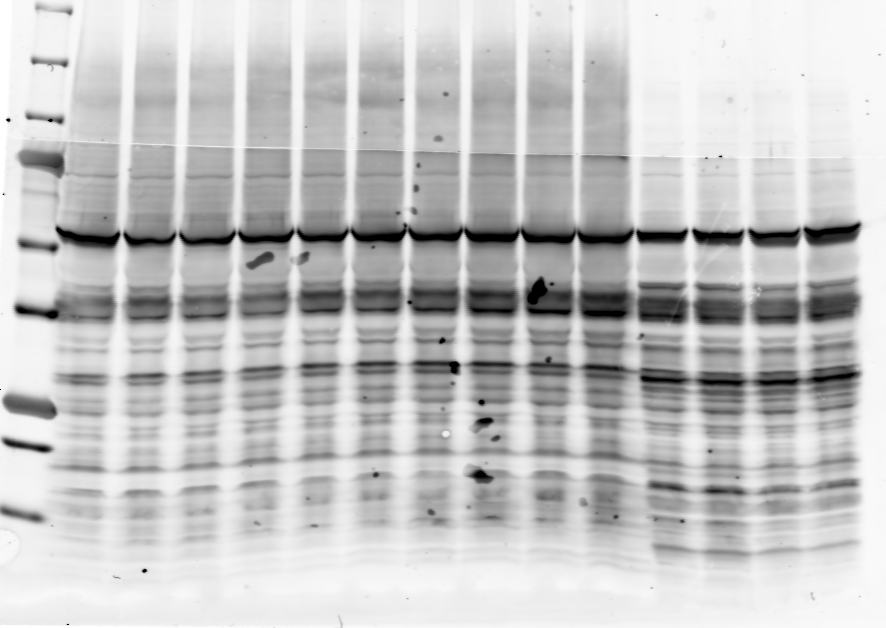

Supplement: Source data 3. [file elife-74531-data3.zip › Source Data - Figure supplements/Source Data Figure 3-figure supplement 1G/Fig 3-FS1G HXK.tif]

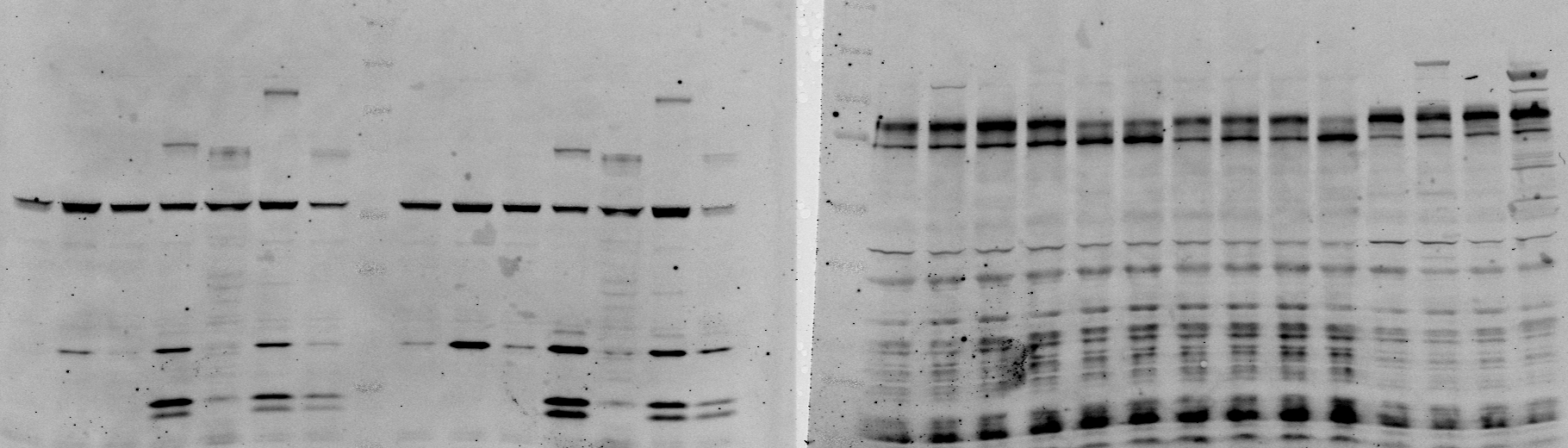

Supplement: Source data 3. [file elife-74531-data3.zip › Source Data - Figure supplements/Source Data Figure 3-figure supplement 1G/Fig 3-FS1G MYC.TIF]

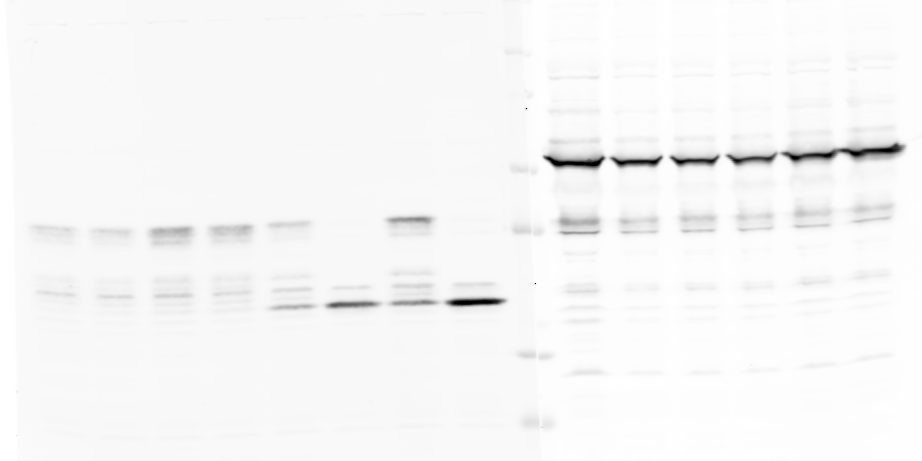

Supplement: Source data 3. [file elife-74531-data3.zip › Source Data - Figure supplements/Source Data Figure 1-figure supplement 1B/Fig 1-FS1B HXK.TIF]

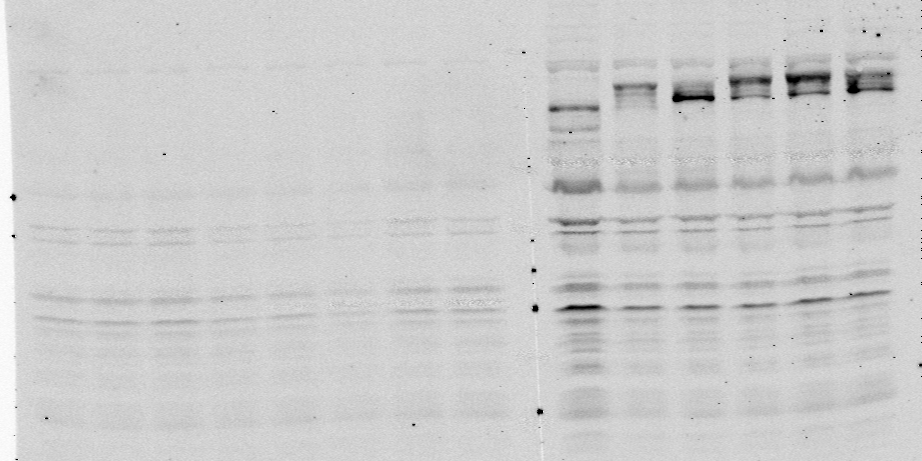

Supplement: Source data 3. [file elife-74531-data3.zip › Source Data - Figure supplements/Source Data Figure 1-figure supplement 1B/Fig 1-FS1B MYC.TIF]

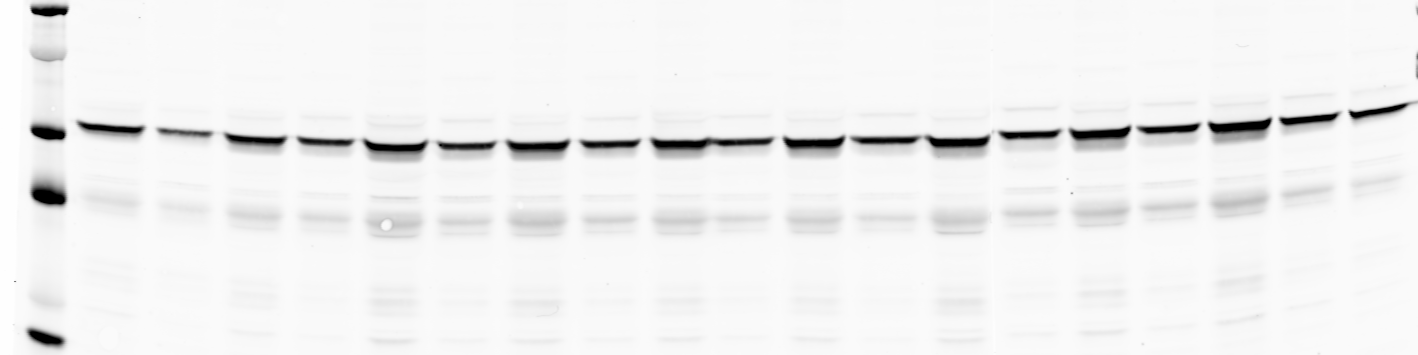

Supplement: Source data 3. [file elife-74531-data3.zip › Source Data - Figure supplements/Source Data Figure 4-figure supplement 1E/Fig 4-FS1E HXK.TIF]

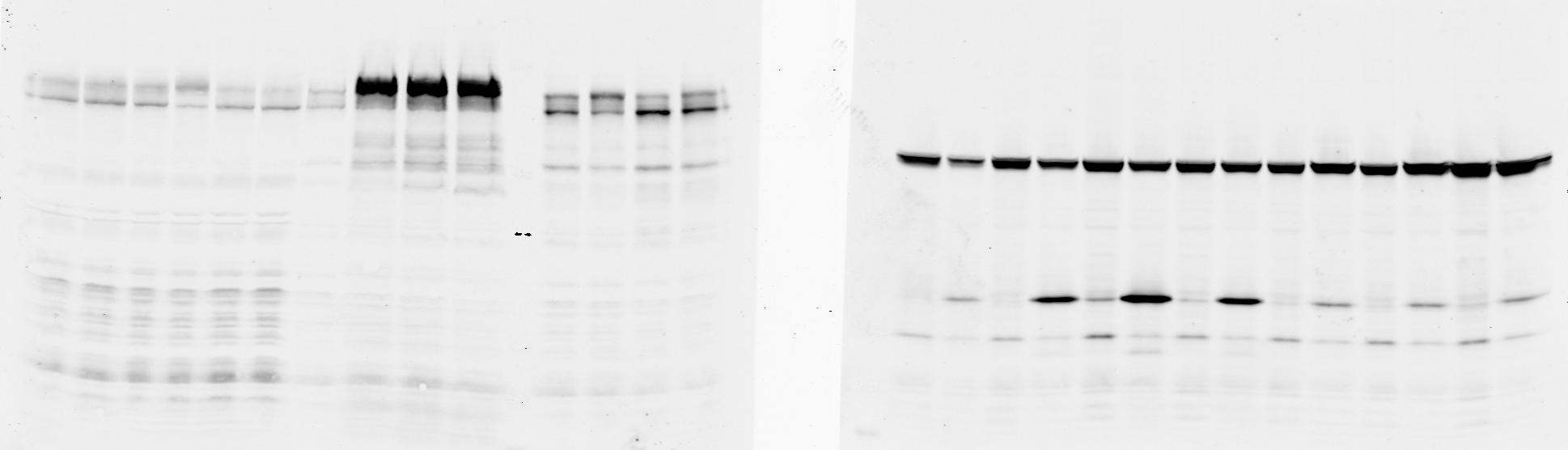

Supplement: Source data 3. [file elife-74531-data3.zip › Source Data - Figure supplements/Source Data Figure 4-figure supplement 1E/Fig 4-FS1E GFP.TIF]

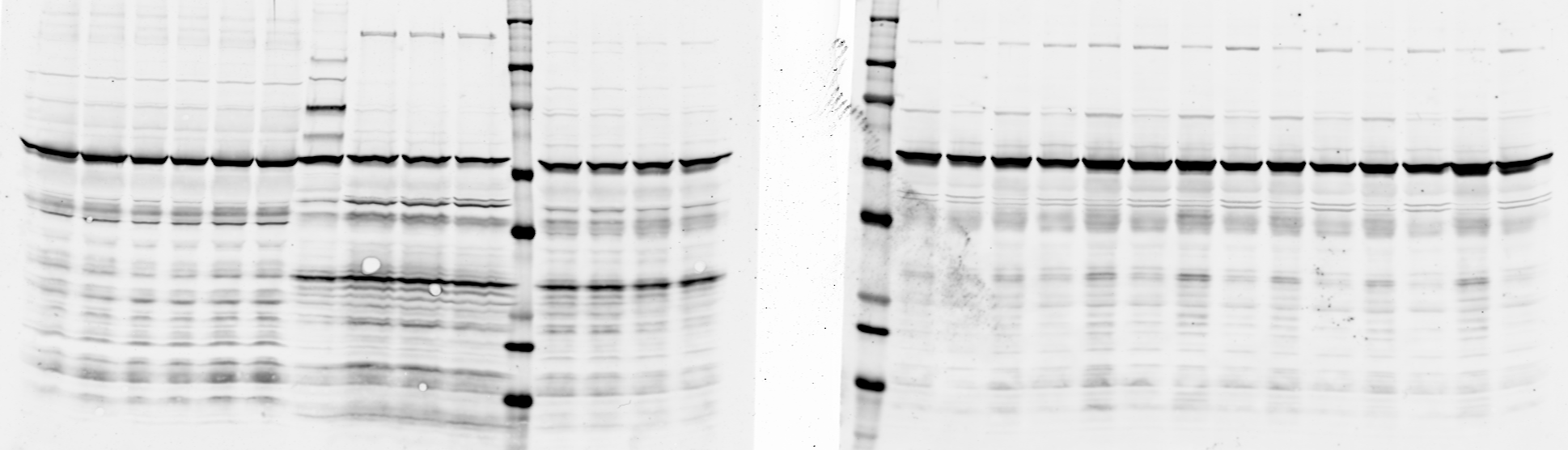

Supplement: Source data 3. [file elife-74531-data3.zip › Source Data - Figure supplements/Source Data Figure 4-figure supplement 1E/Fig 4-FS1E HXK-2.tif]

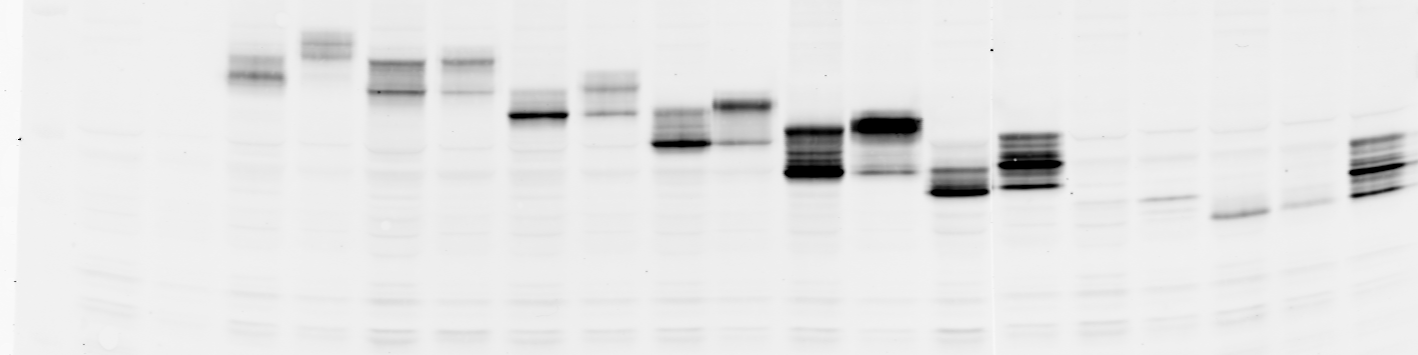

Supplement: Source data 3. [file elife-74531-data3.zip › Source Data - Figure supplements/Source Data Figure 4-figure supplement 1E/Fig 4-FS1E MYC.TIF]

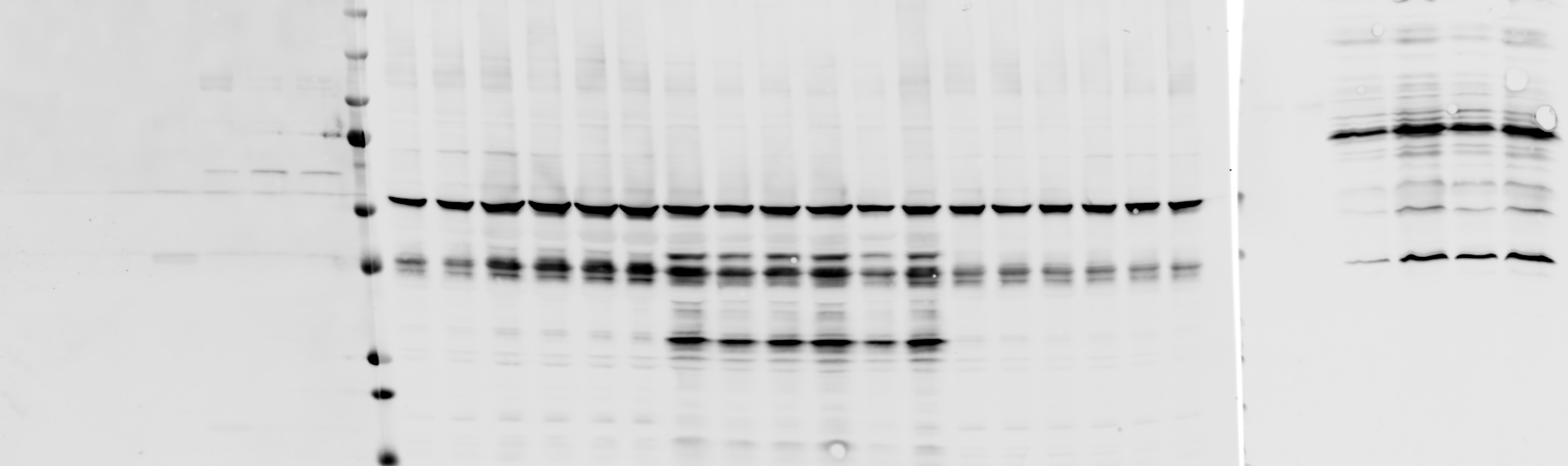

Supplement: Source data 3. [file elife-74531-data3.zip › Source Data - Figure supplements/Source Data Figure 4-figure supplement 1D/Fig 4-FS1D HXK.TIF]

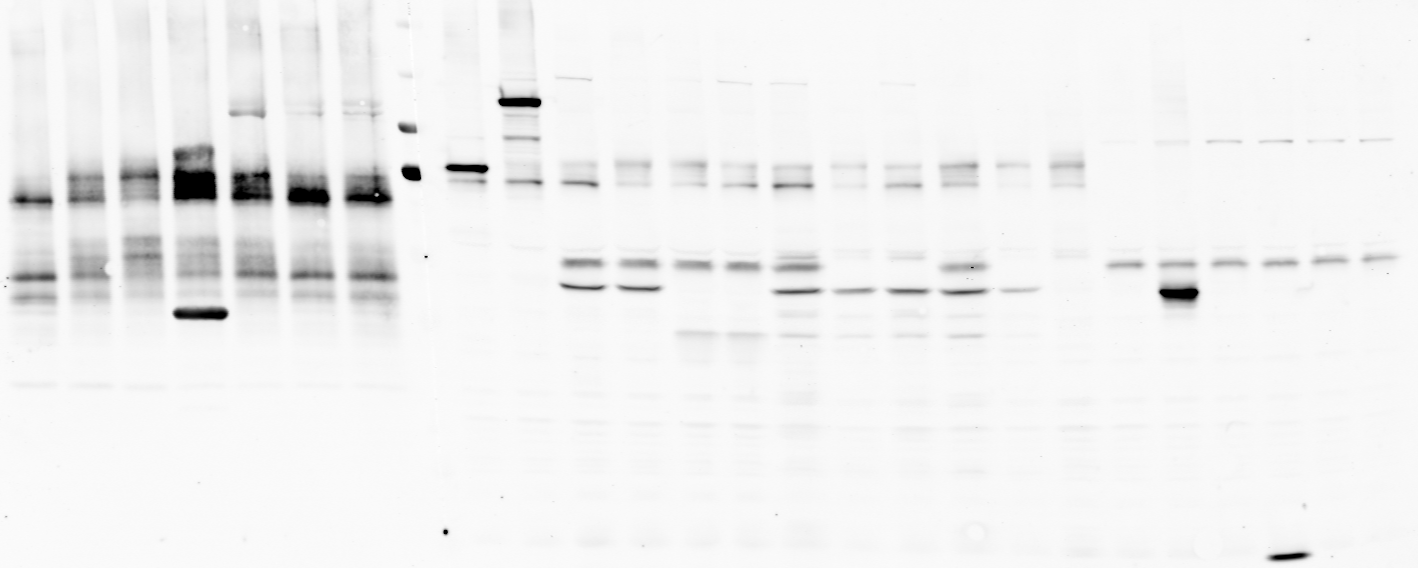

Supplement: Source data 3. [file elife-74531-data3.zip › Source Data - Figure supplements/Source Data Figure 4-figure supplement 1D/Fig 4-FS1D FLAG.TIF]
