# Supplementary material for: The peroxisomal exportomer directly inhibits phosphoactivation of the pexophagy receptor Atg36 to suppress pexophagy in yeast
Source: eLife. 2022 Apr 11;11:e74531. doi: 10.7554/eLife.74531 (PMC9000956; doi:10.7554/eLife.74531)
Supplement: Supplementary file 1. [file elife-74531-supp1.docx]

**Supplementary File 1. Table of yeast strains used in this study**

| **Figure Panel** | **Strain Number** | **Strain Genotype** | **Source or reference** |
| --- | --- | --- | --- |
| 1A | BY4741 | *MATa ura3∆0 his3∆1 leu2∆0 met15∆0* | Standard strain |
| 1A | VDY2603 | *BY4741 ATG36-13×MYC::HIS* | This study |
| 1A | VDY2604 | *BY4741 ATG36-13×MYC::HIS pex1∆::KAN* | This study |
| 1B | VDY2738 | *BY4741 PEX3-3×FLAG::NAT ATG36-13×MYC::HIS PEX1-3×V5-AID::KAN leu2∆::GPDpr-OsTIR1::LEU* | This study |
| 1B | VDY2662 | *BY4741 PEX3-3×FLAG::NAT ATG36-13×MYC::HIS pex1∆::KAN* | This study |
| 1B | VDY2817 | *BY4741 PEX3-3×FLAG::NAT ATG36-13×MYC::HIS PEX1-3×V5-AID::KAN leu2∆::GPDpr-OsTIR1::LEU pex6∆::URA* | This study |
| 1B | VDY2754 | *BY4741 PEX3-3×FLAG::NAT ATG36-13×MYC::HIS PEX1-3×V5-AID::KAN leu2∆::GPDpr-OsTIR1::LEU pex15∆::URA* | This study |
| 1B | VDY2663 | *BY4741 pex3∆::PEX3_177-3×FLAG::NAT ATG36-13×MYC::HIS pex1∆::KAN* | This study |
| 1C | VDY2632 | *BY4741 ATG36-13×MYC::HIS PEX1-3×V5-AID::KAN leu2∆::GPDpr-OsTIR1::LEU* | This study |
| 1C | VDY2633 | *BY4741 ATG36-13×MYC::HIS PEX1-3×V5-AID::KAN HRR25-3×V5-AID::URA leu2∆::GPDpr-OsTIR1::LEU* | This study |
| 1D | VDY5861 | *BY4741 ATG36-13×MYC::HIS PEX11-GFP::HPH pex6∆::PEX6(WB)* | This study |
| 1D | VDY5860 | *BY4741 ATG36-13×MYC::HIS PEX11-GFP::HPH pex6∆::URA* | This study |
| 1D | VDY5859 | *BY4741 ATG36-13×MYC::HIS PEX11-GFP::HPH* | This study |
| 2A,D | VDY2604 | *BY4741 ATG36-13×MYC::HIS pex1∆::KAN* | This study |
| 2A,D | VDY2682 | *BY4741 ATG36-13×MYC::HIS PEX3-3×FLAG::NAT pex8∆::LEU* | This study |
| 2A,D | VDY2683 | *BY4741 ATG36-13×MYC::HIS PEX3-3×FLAG::NAT pex8∆::LEU pex1∆::KAN* | This study |
| 2B | VDY2603 | *BY4741 ATG36-13×MYC::HIS* | This study |
| 2B | VDY2661 | *BY4741 PEX3-3×FLAG::NAT ATG36-13×MYC::HIS* | This study |
| 2B | VDY2684 | *BY4741 PEX3-3×FLAG::NAT ATG36-13×MYC::HIS PEX1-3×V5-AID::KAN* | This study |
| 2C | VDY2632 | *BY4741 ATG36-13×MYC::HIS PEX1-3×V5-AID::KAN leu2∆::GPDpr-OsTIR1::LEU* | This study |
| 2C | VDY2738 | *BY4741 PEX3-3×FLAG::NAT ATG36-13×MYC::HIS PEX1-3×V5-AID::KAN leu2∆::GPDpr-OsTIR1::LEU* | This study |
| 2C | VDY2817 | *BY4741 PEX3-3×FLAG::NAT ATG36-13×MYC::HIS PEX1-3×V5-AID::KAN leu2∆::GPDpr-OsTIR1::LEU pex6∆::URA* | This study |
| 2C | VDY3670 | *BY4741 PEX3-3×FLAG::NAT ATG36-13×MYC::HIS PEX1-3×V5-AID::KAN leu2∆::GPDpr-OsTIR1::LEU pex6∆::PEX6(WB)* | This study |
| 3A | VDY2738 | *BY4741 PEX3-3×FLAG::NAT ATG36-13×MYC::HIS PEX1-3×V5-AID::KAN leu2∆::GPDpr-OsTIR1::LEU* | This study |
| 3A | VDY3656 | *BY4741 PEX3-3×FLAG::NAT ATG36-13×MYC::HIS PEX1-3×V5-AID::KAN leu2∆::GPDpr-OsTIR1::LEU pex5∆::URA* | This study |
| 3A | VDY3657 | *BY4741 PEX3-3×FLAG::NAT ATG36-13×MYC::HIS PEX1-3×V5-AID::KAN leu2∆::GPDpr-OsTIR1::LEU pex14∆::URA* | This study |
| 3A | VDY2755 | *BY4741 PEX3-3×FLAG::NAT ATG36-13×MYC::HIS PEX1-3×V5-AID::KAN pex19∆::LEU* | This study |
| 3B | VDY2632 | *BY4741 ATG36-13×MYC::HIS PEX1-3×V5-AID::KAN leu2∆::GPDpr-OsTIR1::LEU* | This study |
| 3B | VDY2684 | *BY4741 PEX3-3×FLAG::NAT ATG36-13×MYC::HIS PEX1-3×V5-AID::KAN* | This study |
| 3B | VDY2753 | *BY4741 PEX3-3×FLAG::NAT ATG36-13×MYC::HIS PEX1-3×V5-AID::KAN pex14∆::URA leu2∆::GPDpr-OsTIR1::LEU* | This study |
| 3B | VDY2754 | *BY4741 PEX3-3×FLAG::NAT ATG36-13×MYC::HIS PEX1-3×V5-AID::KAN pex15∆::URA leu2∆::GPDpr-OsTIR1::LEU* | This study |
| 3B | VDY2755 | *BY4741 PEX3-3×FLAG::NAT ATG36-13×MYC::HIS PEX1-3×V5-AID::KAN pex19∆::LEU* | This study |
| 3C | VDY2738 | *BY4741 PEX3-3×FLAG::NAT ATG36-13×MYC::HIS PEX1-3×V5-AID::KAN leu2∆::GPDpr-OsTIR1::LEU* | This study |
| 3C | VDY2750 | *BY4741 pex3∆::URA ATG36-13×MYC::HIS PEX1-3×V5-AID::KAN leu2∆::GPDpr-OsTIR1::LEU* | This study |
| 3C | VDY2767 | *BY4741 pex3∆::cytoPEX3-3×FLAG::NAT ATG36-13×MYC::HIS PEX1-3×V5-AID::KAN leu2∆::GPDpr-OsTIR1::LEU* | This study |
| 3C | VDY3426 | *BY4741 pex3∆::cytoPEX15-cytoPEX3-3×FLAG::NAT ATG36-13×MYC::HIS PEX1-3×V5-AID::KAN leu2∆::GPDpr-OsTIR1::LEU* | This study |
| 3D | VDY3676 | *BY4741 ATG36-13×MYC::HIS PEX1-3×V5-AID::KAN leu2∆::GPDpr-OsTIR1::LEU pex15∆::URA pex19∆::HPH* | This study |
| 3D | VDY3677 | *BY4741 pex3∆::cytoPEX15-cytoPEX3-3×FLAG::NAT ATG36-13×MYC::HIS PEX1-3×V5-AID::KAN leu2∆::GPDpr-OsTIR1::LEU pex15∆::URA pex19∆::HPH* | This study |
| 3D | VDY3678 | *BY4741 pex3∆::cytoPEX15-cytoPEX3-3×FLAG::NAT ATG36-13×MYC::HIS PEX1-3×V5-AID::KAN leu2∆::GPDpr-OsTIR1::LEU pex15∆::URA pex19∆::HPH pex6∆::PEX6(WB)* | This study |
| 3E | VDY3504 | *W303 MATa fpr1∆::NAT tor1-1 PEX3-FRB-3×HA::HIS ATG36-13×MYC::KAN pex15∆::TRP1 pex6∆:3×FLAG-FKBP-PEX6* | This study |
| 3E | VDY3511 | *W303 MATa fpr1∆::NAT tor1-1 PEX3-FRB-3×HA::HIS ATG36-13×MYC::KAN pex15∆::TRP1 pex6∆::3×FLAG-FKBP-PEX6(WB)* | This study |
| 4C | VDY5859 | *BY4741 PEX11-GFP::HPH ATG36-13×MYC::HIS* | This study |
| 4C | VDY5862 | *BY4741 PEX11-GFP::HPH atg36∆::URA* | This study |
| 4C | VDY5863 | *BY4741 PEX11-GFP::HPH atg36∆::∆N30atg36-13xMYC::HIS PEX1-3×V5-AID::KAN leu2∆::GPDpr-OsTIR1::LEU* | This study |
| 4C | VDY5864 | *BY4741 PEX11-GFP::HPH atg36∆::Atg36∆30-13xMYC::HIS PEX1-3×V5-AID::KAN leu2∆::GPDpr-OsTIR1::LEU atg11∆::URA* | This study |
| 4C | VDY5865 | *BY4741 PEX11-GFP::HPH atg36∆::PEX15N-∆N30atg36-13xMYC::HIS PEX1-3×V5-AID::KAN leu2∆::GPDpr-OsTIR1::LEU* | This study |
| 4C | VDY5866 | *BY4741 PEX11-GFP::HPH atg36∆::PEX15N(L22S)-∆N30atg36-13xMYC::HIS PEX1-3×V5-AID::KAN leu2∆::GPDpr-OsTIR1::LEU* | This study |
| Figure 1-figure supplement 1A | VDY5859 | *BY4741 Pex11-GFP::HPH ATG36-13×MYC::HIS* | This study |
| Figure 1-figure supplement 1A | VDY5920 | *BY4741 Pex11-GFP::HPH pex1∆::KAN* | This study |
| Figure 1-figure supplement 1A | VDY5921 | *BY4741 Pex11-GFP::HPH pex1∆::KAN atg11∆::LEU* | This study |
| Figure 1-figure supplement 1A | VDY5922 | *BY4741 Pex11-GFP::HPH atg36∆::URA pex1∆::KAN* | This study |
| Figure 1-figure supplement 1A | VDY5923 | *BY4741 Pex11-GFP::HPH ATG36-13×MYC::HIS hrr25∆::HRR25(as) pex1∆::KAN* | This study |
| Figure 1-figure supplement 1B | VDY2603 | *BY4741 ATG36-13×MYC::HIS* | This study |
| Figure 1-figure supplement 1B | VDY2604 | *BY4741 ATG36-13×MYC::HIS pex1∆::KAN* | This study |
| Figure 1-figure supplement 1B | VDY2726 | *BY4741 ATG36-13×MYC::HIS pex6∆::URA* | This study |
| Figure 1-figure supplement 1B | VDY2730 | *BY4741 ATG36-13×MYC::HIS pex15∆::URA* | This study |
| Figure 3-figure supplement 1A | VDY3426 | *BY4741 pex3∆::cytoPEX15-cytoPEX3-3×FLAG::NAT ATG36-13×MYC::HIS PEX1-3×V5-AID::KAN leu2∆::GPDpr-OsTIR1::LEU* | This study |
| Figure 3-figure supplement 1A | VDY3514 | *BY4741 pex3∆::cytoPEX15-cytoPEX3-3×FLAG::NAT ATG36-13×MYC::HIS PEX1-3×V5-AID::KAN leu2∆::GPDpr-OsTIR1::LEU pex19∆::HPH* | This study |
| Figure 3-figure supplement 1B | VDY3426 | *BY4741 pex3∆::cytoPEX15-cytoPEX3-3×FLAG::NAT ATG36-13×MYC::HIS PEX1-3×V5-AID::KAN leu2∆::GPDpr-OsTIR1::LEU* | This study |
| Figure 3-figure supplement 1B | VDY3477 | *BY4741 pex3∆::cytoPEX15-cytoPEX3-3×FLAG::NAT ATG36-13×MYC::HIS PEX1-3×V5-AID::KAN leu2∆::GPDpr-OsTIR1::LEU pex6∆::URA* | This study |
| Figure 3-figure supplement 1B | VDY3478 | *BY4741 pex3∆::cytoPEX15-cytoPEX3-3×FLAG::NAT ATG36-13×MYC::HIS PEX1-3×V5-AID::KAN leu2∆::GPDpr-OsTIR1::LEU pex6∆::PEX6(WB)* | This study |
| Figure 3-figure supplement 1C, D, E | VDY5924 | *BY4741 ATG36-13×MYC::HIS PEX11-GFP::HPH pex3∆::pex3-177-3×FLAG::NAT* | This study |
| Figure 3-figure supplement 1C, D, E | VDY5925 | *BY4741 ATG36-13×MYC::HIS PEX11-GFP::HPH pex3∆::pex3-177-3×FLAG::NAT trp1∆::KAN::TDH3pr-Pex3-mCherry* | This study |
| Figure 3-figure supplement 1C, D, E | VDY5926 | *BY4741 ATG36-13×MYC::HIS PEX11-GFP::HPH pex3∆::pex3-177-3×FLAG::NAT trp1∆::KAN::TDH3pr-cytoPex3-mCherry* | This study |
| Figure 3-figure supplement 1C, D, E | VDY5927 | *BY4741 ATG36-13×MYC::HIS PEX11-GFP::HPH pex3∆::pex3-177-3×FLAG::NAT trp1∆::KAN::TDH3pr-cytoPex15-cytoPex3-mCherry* | This study |
| Figure 3-figure supplement 1E | VDY5859 | *BY4741 ATG36-13×MYC::HIS PEX11-GFP::HPH* | This study |
| Figure 3-figure supplement 1F,G | VDY3499 | *W303 MATa fpr1∆::NAT tor1-1 ATG36-13×MYC::KAN* | This study |
| Figure 3-figure supplement 1F | VDY3501 | *W303 MATa fpr1∆::NAT tor1-1 ATG36-13×MYC::KAN PEX3-FRB-3×HA::HIS* | This study |
| Figure 3-figure supplement 1F | VDY3506 | *W303 MATa fpr1∆::NAT tor1-1 ATG36-13×MYC::KAN pex6∆::3×FLAG-FKBP-PEX6* | This study |
| Figure 3-figure supplement 1F | VDY3500 | *W303 MATa fpr1∆::NAT tor1-1 ATG36-13×MYC::KAN pex15∆::TRP1* | This study |
| Figure 3-figure supplement 1F | VDY3512 | *W303 MATa fpr1∆::NAT tor1-1 ATG36-13×MYC::KAN PEX3-FRB-3×HA::HIS pex6∆::3×FLAG-FKBP-PEX6* | This study |
| Figure 3-figure supplement 1F,G | VDY3504 | *W303 MATa fpr1∆::NAT tor1-1 ATG36-13×MYC::KAN PEX3-FRB-3×HA::HIS pex15∆::TRP1 pex6∆::3×FLAG-FKBP-PEX6* | This study |
| Figure 3-figure supplement 1F,G | VDY3511 | *W303 MATa fpr1∆::NAT tor1-1 ATG36-13×MYC::KAN PEX3-FRB-3×HA::HIS pex15∆::TRP1 pex6∆::3×FLAG-FKBP-PEX6(WB)* | This study |
| Figure 3-figure supplement 1G | VDY5928 | *W303 MATa fpr1∆::NAT tor1-1 ATG36-13×MYC::KAN PEX3-FRB-3×HA::HIS pex15∆::TRP1 trp1∆::CgLEU2::ZE4V-ZDpr- FLAG-Cdc48* | This study |
| Figure 3-figure supplement 1G | VDY5929 | *W303 MATa fpr1∆::NAT tor1-1 ATG36-13×MYC::KAN PEX3-FRB-3×HA::HIS pex15∆::TRP1 trp1∆::CgLEU2::ZE4V-ZDpr-FLAG-FKBP-Cdc48* | This study |
| Figure 4-figure supplement 1B | VDY5867 | *BY4741 PEX11-mCHERRY::HPH ATG36-NEONGREEN::HIS* | This study |
| Figure 4-figure supplement 1B | VDY5868 | *BY4741 PEX11-mCHERRY::HPH atg36∆::atg36(150-293)-NEONGREEN::HIS* | This study |
| Figure 4-figure supplement 1B | VDY5869 | *BY4741 PEX11-mCHERRY::HPH atg36∆::atg36(181-293)-NEONGREEN::HIS* | This study |
| Figure 4-figure supplement 1B | VDY5870 | *BY4741 PEX11-mCHERRY::HPH atg36∆::atg36(1-219)-NEONGREEN::HIS* | This study |
| Figure 4-figure supplement 1B | VDY5871 | *BY4741 PEX11-mCHERRY::HPH atg36∆::atg36(1-180)-NEONGREEN::HIS* | This study |
| Figure 4-figure supplement 1B | VDY5872 | *BY4741 PEX11-mCHERRY::HPH atg36∆::atg36(150-219)-NEONGREEN::HIS* | This study |
| Figure 4-figure supplement 1C, 1D | VDY5873 | *BY4741 PEX11-mCHERRY::HPH ATG36-NEONGREEN::HIS Pex3-3xFLAG::NAT his3∆::CgLEU2::ZE4V* | This study |
| Figure 4-figure supplement 1E | VDY5859 | *BY4741 PEX11-GFP::HPH ATG36-13×MYC::HIS* | This study |
| Figure 4-figure supplement 1E | VDY5862 | *BY4741 PEX11-GFP::HPH atg36∆::URA* | This study |
| Figure 4-figure supplement 1E | VDY5863 | *BY4741 PEX11-GFP::HPH atg36∆::∆N30atg36-13xMYC::HIS PEX1-3×V5-AID::KAN leu2∆::GPDpr-OsTIR1::LEU* | This study |
| Figure 4-figure supplement 1E | VDY5874 | *BY4741 PEX11-GFP::HPH atg36∆::∆N59atg36-13xMYC::HIS PEX1-3×V5-AID::KAN leu2∆::GPDpr-OsTIR1::LEU* | This study |
| Figure 4-figure supplement 1E | VDY5875 | *BY4741 PEX11-GFP::HPH atg36∆::∆N89atg36-13xMYC::HIS PEX1-3×V5-AID::KAN leu2∆::GPDpr-OsTIR1::LEU* | This study |
| Figure 4-figure supplement 1F | VDY5930 | *BY4741 PEX11-GFP::HPH Atg36-13xMYC::HIS PEX3-3×FLAG::NAT* | This study |
| Figure 4-figure supplement 1F | VDY5931 | *BY4741 PEX11-GFP::HPH atg36∆::∆N30atg36-13xMYC::HIS PEX3-3×FLAG::NAT* | This study |
| Figure 4-figure supplement 1F | VDY5932 | *BY4741 PEX11-GFP::HPH atg36∆::PEX15N-∆N30atg36-13xMYC::HIS PEX3-3×FLAG::NAT* | This study |
| Figure 4-figure supplement 1F | VDY5933 | *BY4741 PEX11-GFP::HPH atg36∆::PEX15N(L22S)-∆N30atg36-13xMYC::HIS PEX3-3×FLAG::NAT* | This study |
