# Supplementary material for: The peroxisomal exportomer directly inhibits phosphoactivation of the pexophagy receptor Atg36 to suppress pexophagy in yeast
Source: eLife. 2022 Apr 11;11:e74531. doi: 10.7554/eLife.74531 (PMC9000956; doi:10.7554/eLife.74531)

Figue 3-FS1A

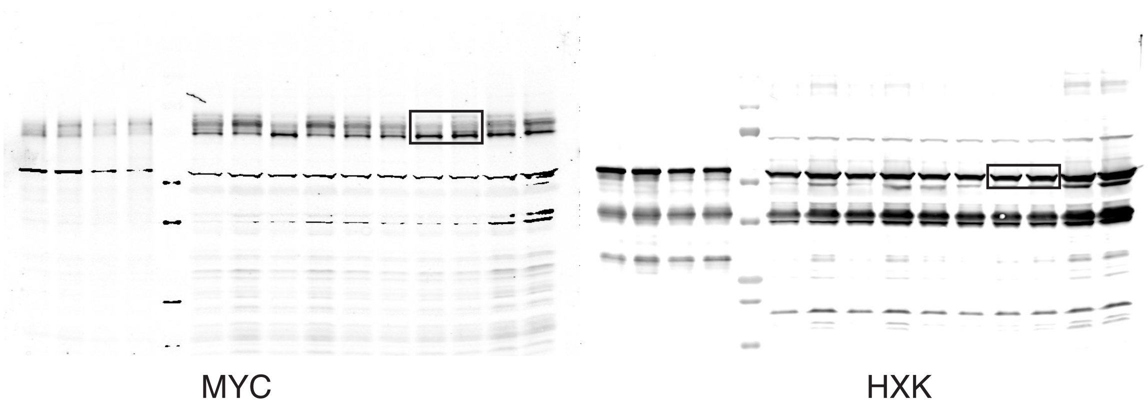

Figue 3-FS1B

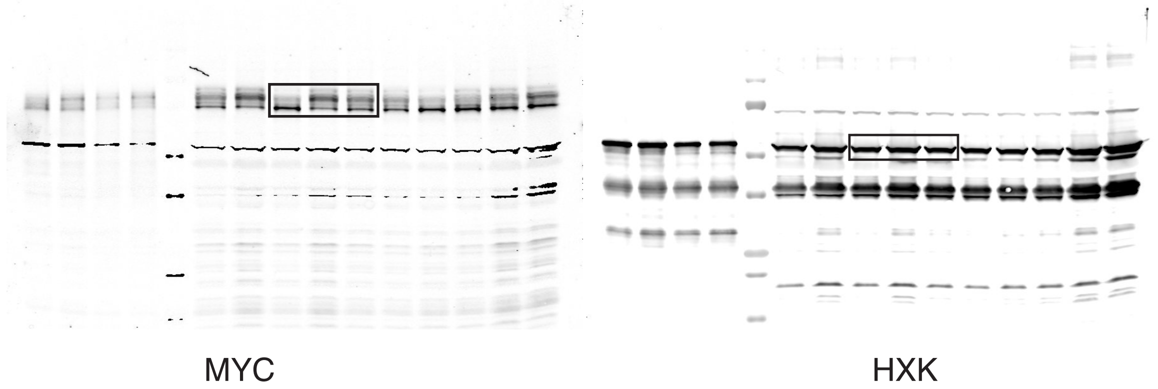

Figue 3-FS1C

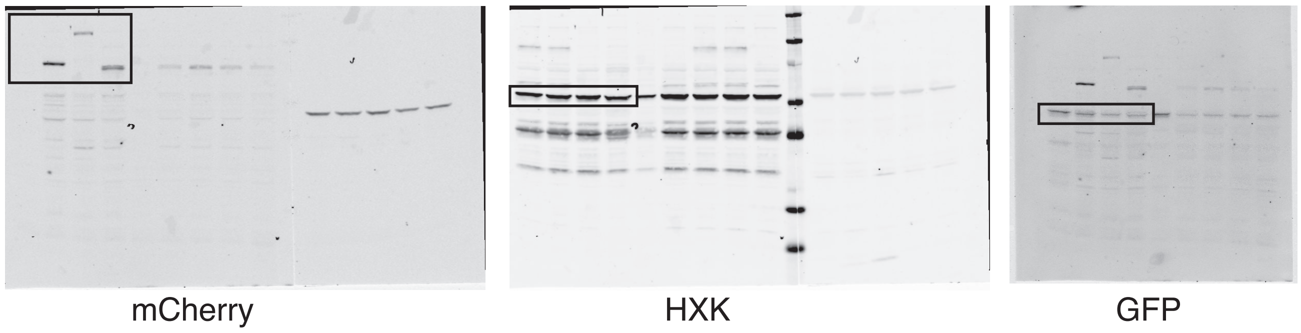

Figue 3-FS1E

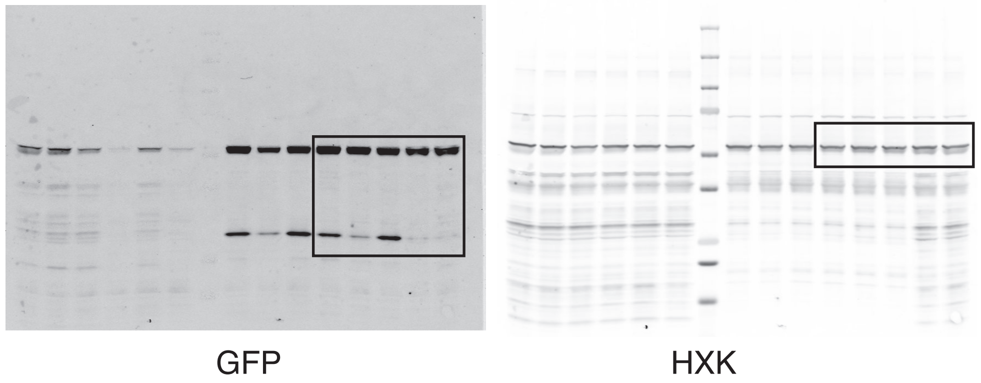

Figue 3-FS1F

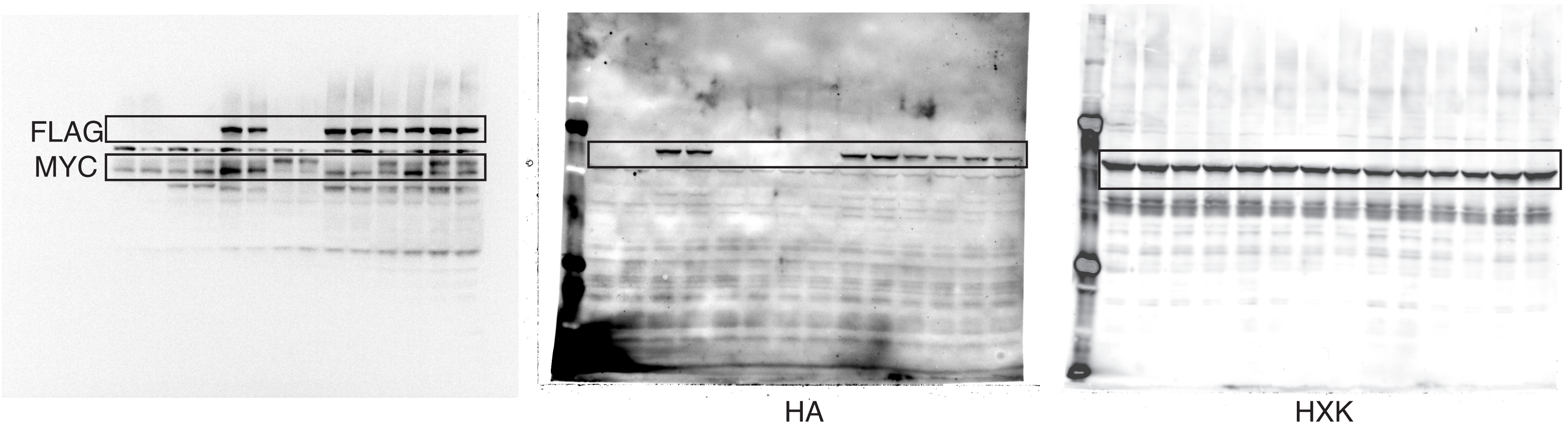

Figue 3-FS1G

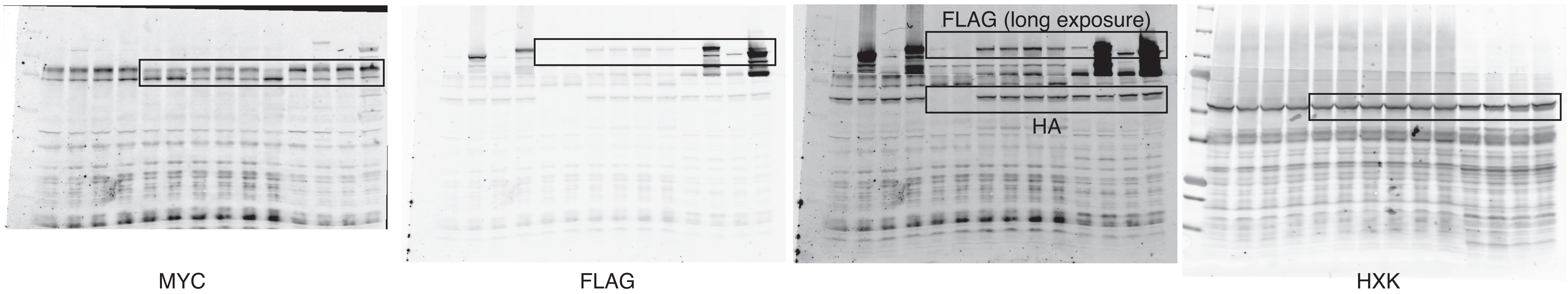

Supplement: Source data 1. [file elife-74531-data1.zip › source data/source data Figure 3-figure supplement 1.pdf]
