# Supplementary material for: The peroxisomal exportomer directly inhibits phosphoactivation of the pexophagy receptor Atg36 to suppress pexophagy in yeast
Source: eLife. 2022 Apr 11;11:e74531. doi: 10.7554/eLife.74531 (PMC9000956; doi:10.7554/eLife.74531)

Figure 3A

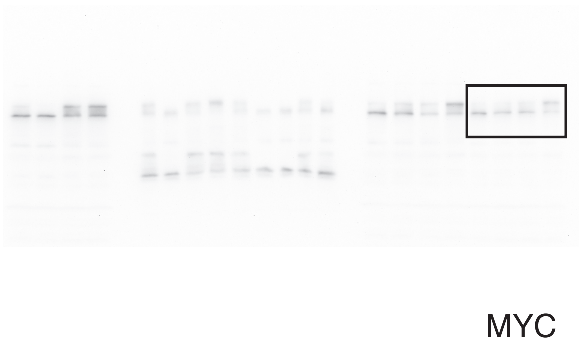

Figure 3B

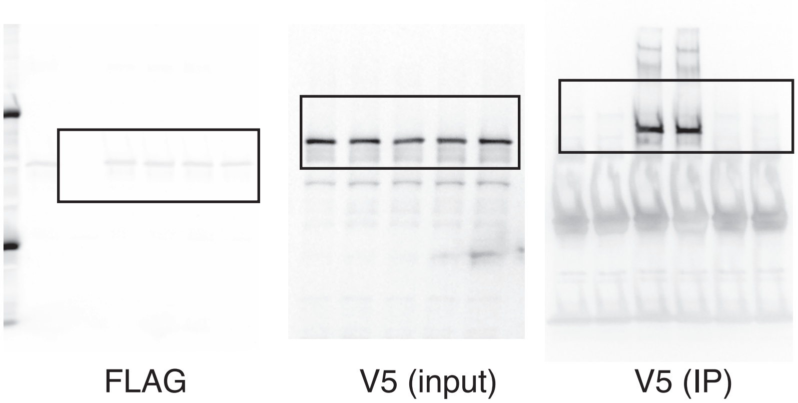

Figure 3C

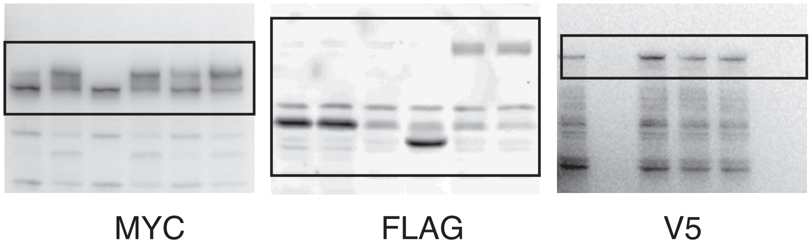

Figure 3E

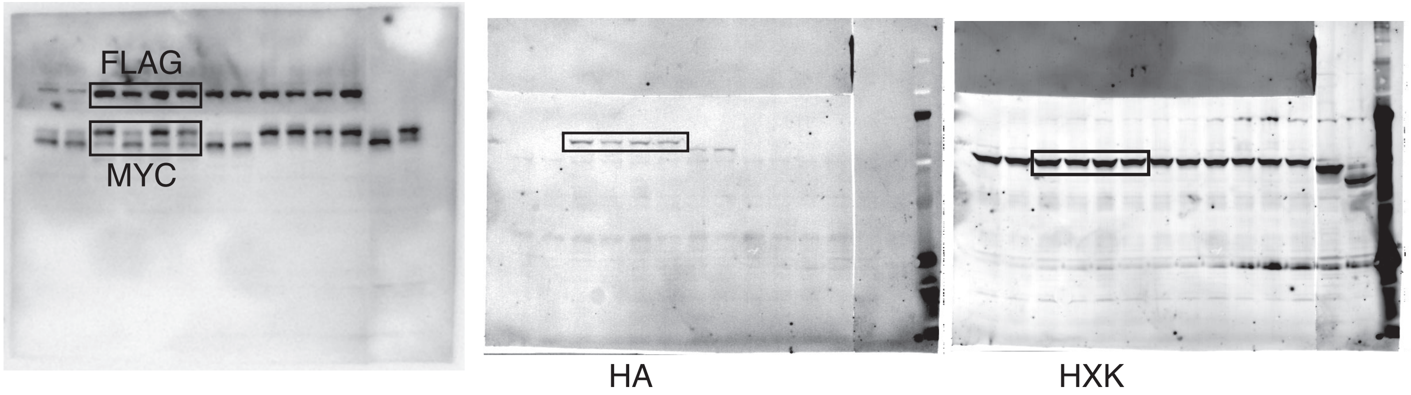

Figure 3F

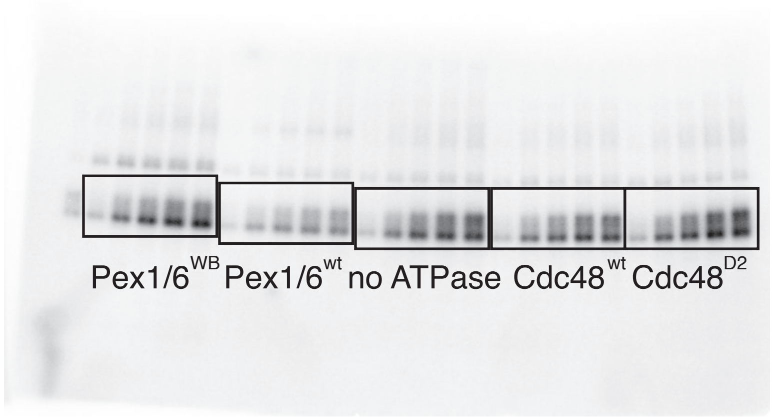

Figure 3G

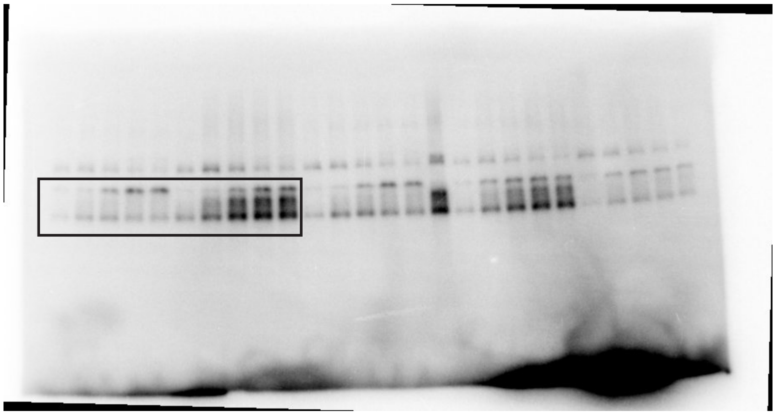

Supplement: Source data 1. [file elife-74531-data1.zip › source data/source data Figure 3.pdf]
